# A Human Embryonic Stem Cell Model of Aβ-Dependent Chronic Progressive Neurodegeneration

**DOI:** 10.3389/fnins.2019.01007

**Published:** 2019-09-20

**Authors:** Teresa Ubina, Martha Magallanes, Saumya Srivastava, Charles D. Warden, Jiing-Kuan Yee, Paul M. Salvaterra

**Affiliations:** ^1^Department of Developmental and Stem Cell Biology, Beckman Research Institute – City of Hope, Duarte, CA, United States; ^2^Department of Biology, California State University, San Bernardino, San Bernardino, CA, United States; ^3^Integrative Genomics Core, Beckman Research Institute – City of Hope, Duarte, CA, United States; ^4^Department of Diabetes, Beckman Research Institute – City of Hope, Duarte, CA, United States; ^5^Irell and Manella Graduate School of Biological Sciences, Beckman Research Institute – City of Hope, Duarte, CA, United States

**Keywords:** amyloid-beta, neurodegeneration, stem cells, Alzheimer’s disease, Alzheimer’s model

## Abstract

We describe the construction and phenotypic analysis of a human embryonic stem cell model of progressive Aβ-dependent neurodegeneration (ND) with potential relevance to Alzheimer’s disease (AD). We modified one allele of the normal APP locus to directly express a secretory form of Aβ40 or Aβ42, enabling expression from this edited allele to bypass the normal amyloidogenic APP processing pathway. Following neuronal differentiation, edited cell lines specifically accumulate intracellular aggregated/oligomeric Aβ, exhibit a synaptic deficit, and have an abnormal accumulation of endolysosomal vesicles. Edited cultures progress to a stage of overt ND. All phenotypes appear at earlier culture times for Aβ42 relative to Aβ40. Whole transcriptome RNA-Seq analysis identified 23 up and 70 down regulated genes (differentially expressed genes) with similar directional fold change but larger absolute values in the Aβ42 samples suggesting common underlying pathogenic mechanisms. Pathway/annotation analysis suggested that down regulation of extracellular matrix and cilia functions is significantly overrepresented. This cellular model could be useful for uncovering mechanisms directly linking Aβ to neuronal death and as a tool to screen for new therapeutic agents that slow or prevent human ND.

## Introduction

Despite intensive research efforts, no effective treatment or preventative strategies for Alzheimer’s disease (AD) have yet been identified. Reasons for this include the relative experimental inaccessibility of human brain, a lengthy preclinical symptom free stage, as well as the complexity of the disorder.

Relative inaccessibility has required that most experimental observations are made using non-human AD models. Transgenic rodent models^1^ have been dominant for preclinical studies which have largely failed in human trials (Cummings et al., 2014; Sasaguri et al., 2017). The reasons for these failures are unknown but often attributed to technical explanations such as the stage of AD during treatment, unknown target engagement, improper dosage, or off-target “side effects.” An alternative explanation could be more generally related to well-known phenotypic deficiencies of all current AD models (Ashe and Zahs, 2010; Drummond and Wisniewski, 2017; Sasaguri et al., 2017). For example, most rodent (indeed all animal) AD models do not exhibit tau-related disease phenotypes (Drummond and Wisniewski, 2017). Extensive work has identified critical species-specific differences in tau isoforms that can partially account for this model deficiency (Stancu et al., 2014; Frost et al., 2015). Another common deficiency of most rodent models is the absence of chronic progressive neurodegeneration (ND), arguably the main cellular phenotype of AD. Perhaps species differences could also account for this.

Rodents have different amino acid sequences for APP processing enzymes as well as different amino acid sequences in the Aβ peptides thought to play a key role in initiating AD but still controversial (Musiek and Holtzman, 2015; Karran and De Strooper, 2016; Selkoe and Hardy, 2016). The cell biology of APP processing within neurons is still incompletely defined mechanistically (Vetrivel and Thinakaran, 2006; Haass et al., 2012; Sannerud et al., 2016; Liu et al., 2019), especially with respect to the site of production in human neurons. Also, the forms of Aβ which mediate AD phenotypes are not yet definitively established (Broersen et al., 2010; Mucke and Selkoe, 2012; Walsh and Selkoe, 2016). Some believe that Aβ containing plaques may be the culprit, even though they do not correlate well with other AD clinical phenotypes (Dickson et al., 1995). Oligomers are thought by many to be implicated, but the precise forms are still not well-defined biochemically (Benilova et al., 2012). The relative importance of accumulation of intracellular vs. extracellular Aβ with respect to downstream pathology has also not been clearly established (Oddo et al., 2006; Laferla et al., 2007; Gouras et al., 2010).

With the development of induced pluripotent stem cells (iPSC) technology, it is now possible to produce cell culture models that exhibit several AD-relevant phenotypes including Aβ accumulation in a human genetic context when cells are differentiated into neurons (Young and Goldstein, 2012; Mungenast et al., 2016; Arber et al., 2017). These cellular models have even been extended to mimic some limited features of brain like organization using 3D culture technology (Choi et al., 2014; Lee et al., 2016; Raja et al., 2016). This has resulted in a fuller elaboration of potential tau-related phenotypes as well as hypothesized roles for non-neuronal cell types. Unfortunately, current human iPSC models as well as edited iPSC or human embryonic stem cell (hES) models have not yet been shown to progress to a state of overt neuronal death (i.e., ND). Perhaps this could be explained by the limited time cells can be kept in culture relative to the many decades it takes to exhibit AD.

One potential approach to speed up Aβ production is to express peptide coding sequences directly rather than depend on their production through the amyloidogenic APP proteolysis. Amyloidogenic Aβ production is a complex time-dependent process with incompletely defined cell biology and uncertain kinetics (Haass et al., 2012; Rajendran and Annaert, 2012; Toh and Gleeson, 2016). Traditionally, APP amyloidogenic processing was believed to occur primarily or exclusively at the plasma membrane, but more recent studies document intracellular membrane compartments that also produce Aβ (Laferla et al., 2007; Wirths et al., 2012). The relative importance of these different spatially distinct sites with respect to downstream AD phenotypes is still uncertain. Several rodent and invertebrate transgenic models have used direct Aβ expression using a strong promoter to obtain high level (over expression) of the peptides (LaFerla et al., 1995; Finelli et al., 2004; McGowan et al., 2005; Iijima-Ando and Iijima, 2009; Abramowski et al., 2012). All show progressive ND as well as a host of other putative AD-related phenotypes. Interestingly, all of these direct expression models exhibit selective toxicity to Aβ42 but not Aβ40.

In this study, we used transcription activator-like effector nuclease (TALEN) genomic editing to modify one allele of the APP gene to directly express either Aβ40 or Aβ42 in human embryonic stem cells (WiCell Foundation, WA09, aka H9). This experimental design ensures that the peptides will be expressed under control of the normal APP promoter and that the edited cell lines will be isogenic to the unedited cell line. We found that editing did not affect early culture development or neuronal differentiation. All of the AD-like phenotypes we observed can be attributed to the downstream consequences of the early appearance of aggregated/oligomeric Aβ accumulation within neurons. Notably, Aβ42 expressing cell lines elaborate phenotypes at a significantly faster rate than Aβ40 lines even though both edited genes are apparently transcribed at similar levels. Direct Aβ expression resulted in two new potential AD-related phenotypes for human ES models: synaptic deficits and chronic progressive ND (i.e., ND occurs over several months and results in no viable cells for either edited phenotype after 120 days) suggesting that this model could be useful for future investigations into the largely unknown mechanism(s) of human neuronal Aβ-dependent synaptic loss and cell death. Interestingly, RNAseq analysis of gene expression suggests that primary cilia signaling pathways may be involved in initiating a downstream cascade of phenotypes resulting in eventual ND.

## Materials and Methods

### Genomic Editing

Transcription activator-like effector nuclease pairs were designed to target DNA upstream of the normal APP translation start site using published criteria, their cutting efficiency established in HEK293T cells and used to generate a double strand break (DSB) in the APP target (Cermak et al., 2011). Donor templates for homology repair contained homology arms flanking the targeted site along with a secretory signal derived from the rat proenkephalin (PENK) gene, a human Aβ40 or Aβ42 coding sequence, and a polyA tail. Donor templates also contained a puromycin selection gene under control of the human phosphoglycerate kinase.

H9 (WiCell WA09) human embryonic stem cells were obtained from the WiCell Foundation and cultured on a feeder free system (Matrigel). Cells were harvested at appropriate confluency and nucleofected with TALEN pairs and donor template using an Amaxa Nucelofector. Nucleofected cells were grown for 48 h, harvested, and plated on puromycin-resistant feeder cells at a dilution of 1/30 for 48 h and then transferred to puromycin drug selection media for 2 weeks. Approximately 1/2 of appropriate size colonies were collected for PCR analysis using primer pairs that spanned the flanking DNA and the donor plasmid sequences to confirm insertion of the expression cassette. The stem cell colonies positive for correct size PCR fragments at both the 3′- and 5′-sites were expanded and analyzed for expression of edit-specific Aβ40 or Aβ42 expression using qRT-PCR analysis. The forward primer was specific to the rat secretory signal sequence (not present in the human genome) and the reverse primer targets the end of the Aβ40 sequence. The specific sequences and editing and verification details are included in the Supplementary Methods and Data.

### Cell Culture

embryonic stem cell (ESC) culture, embryoid body (EB) generation, and neuronal differentiation were adapted from a well-established protocol (Amoroso et al., 2013). Briefly, stem cells were grown in gelatin-coated six-well plates on an irradiated mouse embryonic fibroblasts feeder layer. Stem cells were maintained in Human embryonic stem (HuES) medium which was replaced daily and differentiating colonies were manually removed to maintain pluripotency. Stem cells were passaged weekly and differentiation was initiated ∼1 week after passage using dissociated cells transferred to a 10-cm culture plate for EB generation. On day 3 cells were grown in Neural Induction Media (NIM) with N2 supplement and 2 μg/ml heparin. On day 5 media was supplemented with ascorbic acid, *trans*-retinoic acid, Y-27632 ROCK inhibitor, and brain-derived neurotrophic factor (BDNF). On day 7 smoothened agonist 1.3 was added. Media was replaced every 3rd day and after ∼28–31 days EBs were collected, rinsed with Ca^2+^-/Mg^2+^-free phosphate-buffered saline (PBS), dissociated into individual cells, and plated in either 6- or 24-well culture plates precoated with poly-L-ornithine and laminin (1.7 × 10^6^ or 0.34 × 10^6^ cells per well) in neural differentiation medium supplemented with 25 μM β-mercaptoethanol and 25 μM glutamate. Cultures were initially treated with 0.5 μM ethynyl deoxyuridine (EdU) for 24 h and weekly thereafter up to ∼50 days to maintain only post-mitotic cells. Complete media recipes, suppliers, and protocol details are included in the Supplementary Methods and Data.

### qRT-PCR

Total RNA was extracted using the RNeasy Micro Kit manufactured by Qiagen following the manufacturer’s protocol. RNA concentration and purity was determined spectrophotometrically and cDNA prepared using qScript cDNA SuperMix (Quanta) following the manufacturers’ protocol. All reactions were carried out in a 20 μl reaction mixture containing 12.5 μl iQTM SYBR Green Supermix (Bio-Rad), 2 μM of each forward and reverse primer, 0.25 μg cDNA, and diethyl pyrocarbonate (DEPC)-Treated Water (Ambion) to adjust the final volume to 20 μl. Amplification was carried out using a BioRad CFX96 Touch^TM^ Real-Time PCR machine in clear 96-well sealed plates, and data were collected and analyzed using BioRad CFX Manager (v3.1). Additional details and primer sequences are included in the Supplementary Methods and Data.

### Microscopy, Immunocytochemistry, Live–Dead Analysis, and Image Analysis

Fluorescence samples were observed with a Zeiss Axio Observer microscope (Xenon illumination) using either a 20× NA = 0.80 plan-apochromat objective or a 40× or 63× plan-apochromat objective (NA = 1.4, Oil). Optical Z sections were acquired with a Zeiss Axiocam506 camera using Zeiss Zen Blue microscope control software (SP2). Unstained cultures were observed using a Nikon Diaphot inverted microscope equipped with Hoffman modulation contrast objectives (HMC EF 10× NA = 0.25 or HMC 20× LWD NA = 0.4) and images were obtained with a SPOT RT230 cooled CCD camera operated by SPOT Advanced Imaging Software. Image analysis used semi or fully automated macros implemented in the FIJI version of NIH ImageJ (v1.46 or 2) (Schindelin et al., 2012). For visual clarity some images are adjusted for brightness and contrast using Adobe Photoshop (CS4 or CS5). Due to variability in the number of cells in neuronal clusters both among genotypes differentiated in parallel, as well as across independent differentiations, quantitative data were usually normalized to the number or area of 4′,6-diamidino-2-phenylindole (DAPI), staining.

### Antibody Staining

Cells were grown on polyornithine-/laminin-coated 15 mm No. 1 glass coverslips (Fisher Scientific) placed in 6-well plates. Cells were fixed with 4% paraformaldehyde for 20 min followed by washing in PBS (3×, 5 min) and coverslips were stored in 0.03% NaN_3_ in PBS at 4°C until observation. Coverslips were incubated with blocking buffer (0.3% Triton X-100 and 5% bovine serum albumin in PBS) for ∼2 h at room temperature, washed briefly with PBS, and incubated overnight at 4°C with primary antibody diluted in 0.3% Triton X-100 and 1% bovine serum albumin in PBS (antibody ilution buffer). Coverslips were washed with PBS (3 × 5 min) with antibody dilution buffer and incubated with fluorescent labeled secondary antibodies for 2 h at room temperature, washed with PBS, incubated with DAPI (1 μg/μl) for 5 min at room temperature, washed with PBS (2×, 5 min), and mounted onto glass slides using DAKO Fluorescent Mounting Medium. Additional coverslips were stained after eliminating either the primary or secondary antibody to serve as negative staining controls. Specific antibody staining details and image analysis parameters are included in the Supplementary Methods and Data.

### Live–Dead Analysis

Neuronal viability was estimated by measuring the relative proportion of live/dead cells in neuronal clusters grown on coverslips or directly in culture wells using a commercial fluorescence assay (ThermoFisher LIVE/DEAD^TM^ Viability/Cytotoxicity Kit, for mammalian cells, #L322) according to the manufacturer’s directions. Additional details and image analysis parameters are included in the Supplementary Methods and Data.

### Statistical Analysis

We used Prism (v7, Graph Pad) for statistical analyses (descriptive statistics, ANOVA, variance estimates, and correlation) and graphic preparation.

### RNA-Seq

Stem cells were differentiated for 36 or 38 days and total RNA was extracted using the RNeasy Micro Kit (Qiagen) following the manufacturer’s protocol. RNA concentration and purity was determined using a NanoDrop ND-1000 spectrophotometer and processed for RNA-Seq analysis by the City of Hope Genomic Core Facility. Detailed processing and analysis protocols are included in the Supplementary Methods and Data. The sequencing data files have been deposited in the NIH GEO database (GSE119527).

## Results

### Model Construction

**FIGURE 1:**
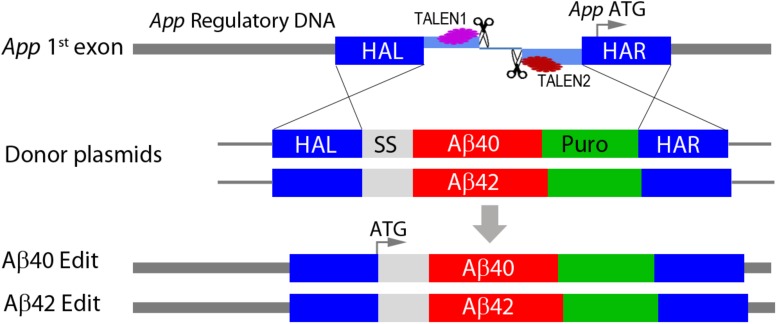
Genomic editing of APP gene locus. TALEN pairs were designed to target and induce a double strand break (DSB) in the first exon upstream of the normal APP translation initiation codon (APP ATG). The DSB was repaired by homologous recombination in the presence of plasmids containing the coding sequence for either Aβ40 or Aβ42 fused in frame with a rat preproenkephalin secretory signal sequence (SS) and followed by a polyA tail. Repair plasmids additionally included a PGK puromycin drug selection gene (Puro) and were flanked by left and right homology arms homologous to APP flanking sequences (HAL, HAR). Cassette insertions were confirmed by genomic PCR using specific primers in either the HAL (5′) or the HAR (3′) and a site in the insertion cassette. This editing strategy simultaneously inactivates one APP allele and replaces it with a cassette that directly expresses a secretory form of either Aβ40 or Aβ42 under normal APP regulatory control. The specific sequences and other details are included in the Supplementary Methods and Data.

We used TALEN genomic editing to modify the normal wild-type APP gene in WiCell WA09 (H9) hES cells. This cell line was chosen because of its widespread use in stem cell studies, the availability of many well characterized neuronal differentiation protocols, and because they contain one e4 allele of APOE (the major genetic risk factor for sporadic AD) (Genin et al., 2011). The APOE genotype (e4/e3) was confirmed using allele-specific PCR analysis. The editing strategy is shown schematically in Figure 1. TALEN pairs were designed to induce a DSB within the first exon of the *App* locus upstream of the normal *App* transcriptional start site. The DSB was repaired by homologous recombination in the presence of donor plasmids that contained a secretory signal sequence derived from the rat preproenkephalin gene (PENK, *Rattus norvegicus*) fused in frame to either a human Aβ40 or Aβ42 coding sequence and followed by a polyA tail just upstream of a puromycin drug selection gene. This insertion cassette was flanked by left and right homology arms to direct insertion into the normal *App* locus.

Successful editing resulted in inactivation of the modified *App* allele and its replacement with direct expression of either secretory Aβ40 or Aβ42. Importantly, the parental and edited cell lines are essentially isogenic ensuring that phenotypic differences are directly attributable to the specific edits. The rat PENK secretory signal sequence is not present in the human genome allowing PCR analysis to specifically detect edited Ab transcripts. Following translation, the signal peptide is completely removed by normal secretory pathway processing resulting in direct production of either an Aβ40 or Aβ42 peptide (Iijima et al., 2004; Abramowski et al., 2012) eliminating any requirement for amyloidogenic APP processing by β and g secretase. Since the edits are introduced directly into the normal APP locus, expression will be under control of the normal APP regulatory DNA. This distinguishes our model from others that generally used exogenous promoters to drive overexpression. We hypothesized that this model could potentially speed up proteotoxic Ab accumulation on a time scale suitable for working with cultured human neurons while potentially minimizing overexpression artifacts.

Proper editing was initially identified by PCR screening of multiple subclones using 3′- and 5′-specific primers and confirmed by genomic sequencing. Since subcloning as well as TALEN editing has the potential to generate off-target effects (primarily indels) or other mutations, although at extremely low levels (Woodruff et al., 2013), we phenotypically characterized two independently isolated subclones for each edited genotype in parallel. We noted no consistent phenotypic differences between subclones suggesting that the differences we describe are genotype-specific (i.e., due to direct expression of either Aβ40 or Aβ42). All edited cell lines used in this study were heterozygous for the edit ensuring that normal APP will still be expressed from the unedited allele.

### Expression

#### qRT-PCR

**FIGURE 2:**
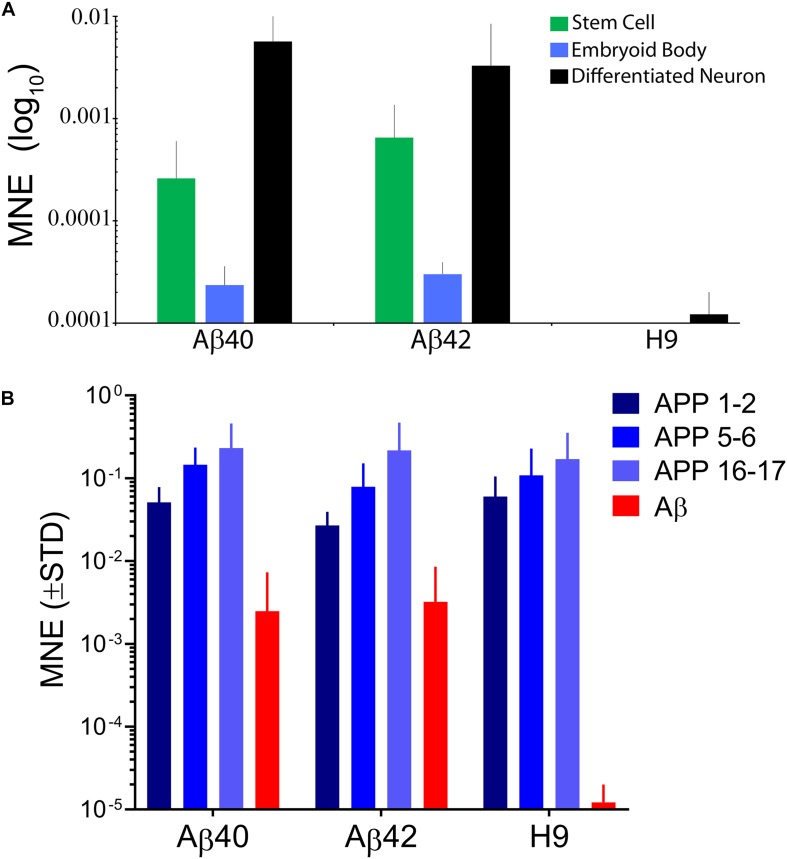
**(A)** Direct expression levels of edit specific Aβ are similar for both edited genotypes and dynamic during early stages of differentiation. Stem cell cultures have intermediate expression, embryoid bodies have significantly less expression and differentiated neurons have the highest, reaching maximal expression by ∼10–20 days after EB dissociation and plating in neural differentiation medium. The relative ratios of Aβ expression were ∼1:0.05:5 for the three developmental stages. There were no significant differences in expression level comparing edit specific Aβ40 and Aβ42 at any stage (ANOVA, Dunnett’s correction). No significant secretory Aβ expression was detected in unedited H9 samples. Data were from 6 independent stem cell cultures, 4 EB stage cultures, and 22 individual 10–20-day-old differentiated neuronal cultures. **(B)** Editing does not affect APP expression from unedited alleles. We used primer pairs spanning three different APP exons. The pattern of expression was similar for all three genotypes and average relative expression for the primer pairs was 1:0.71:0.5 for H9:Aβ40:Aβ42 and is consistent with expected inactivation of one APP due to editing. Expression of edit specific Aβ was ∼30-fold less than APP expression and is replotted from panel **(A)** for comparison. Data were from 4 independent differentiations of H9 cells and 8–20 differentiations for edited genotypes taken from 10- to 34-day-old cultures. In panel **(A)** expression was measured by qRT-PCR using a forward primer specific to the secretory signal sequence (not present in the human genome) and reverse primer to a sequence common to Aβ40 and Aβ42. In panel **(B)** the forward and reverse primers spanned indicated exons in the APP sequence. Bars are mean normalized expression (MNE) relative to GAPDH (±STD).

We used qRT-PCR to measure edit specific expression of secretory tagged Ab. The forward primer was specific to the rat PENK secretory signal peptide which is absent from the human genome and a reverse primer to the end of the Aβ40 sequence which is present in both edits. As expected, no edit specific transcripts were detected in unedited H9 cells (Figure 2A). Significant levels of direct Aβ expression were found in undifferentiated stem cells, EB stage cells, or differentiated neurons. The relative expression levels were similar for both edited genotypes at these three developmental stages indicating that they are under the same transcriptional regulatory control. We additionally confirmed that only secretory tagged Aβ42 expression could be detected in Aβ42-edited lines using a reverse primer specific to the unique 5′-nucleotides in Aβ42. Undifferentiated stem cells show an intermediate expression level, consistent with the normal APP expression pattern previously reported at this stage (Bergström et al., 2016). Transcript abundance decreased significantly during EB formation and increased to the highest levels in 10-day-old neuronally differentiated cultures. The relative ratio of edit-specific Ab mRNA for stem cells, embryoid bodies, and differentiated neurons was ∼20:1:100. We thus expect that Ab protein levels would likely be possibly approximately fivefold greater in differentiated neurons relative to undifferentiated stem cells.

APP expression in 10-day-old differentiated neurons was measured using forward and reverse primers that span different adjacent exons along the length of the normal neuronal APP transcript (Figure 2B). Different exon spanning primer pairs detected APP transcripts over an approximately eightfold range, but the pattern was similar for all three genotypes. The average relative APP expression for all three primer pairs compared to H9 was 0.71 for Aβ40 and 0.5 for Aβ42 a result is consistent with expected inactivation of only the edited APP allele confirming that editing does not drastically affect APP expression from the unedited allele. Unexpectedly, however, we found that direct Ab expression from edited alleles was ∼30-fold lower relative to APP expression (the Ab data are replotted from Figure 2A). This could be due to weakening of a regulatory element in the first intron of APP (Shakes et al., 2012) or alternatively to negative interference of the drug selection gene present in the insertion cassette (Davis et al., 2008). Whatever the reason, direct expression levels for edit-specific Ab are significantly lower than APP.

#### Aβ Protein Analysis

We were unable to measure Aβ protein in either immunoprecipitated culture supernatants (10 ml of immunoprecipitated sample pooled from five samples every 2 days from a single well of a 12-well culture plate), or in guanidine hydrochloride or formic acid cell extracts (prepared from 2 individual 12 well cultures) using commercial ELISA kits (Invitrogen, Aβ40 #KHB3481, sensitivity 6 pg/ml; Aβ42 #KHB3441, sensitivity = 10 pg/ml). Other AD-related human iPSC models were also unable to detect Aβ accumulation in cell extracts using ELISA, possibly indicating a technical limitation of commercial ELISAs (Israel et al., 2012; Muratore et al., 2014). We conclude that Aβ protein levels were below the detection level of the assay since all positive controls were consistent with the manufacturers’ reported sensitivity. These negative results are consistent with our qRT-PCR analysis and suggest that Ab levels in our directly expressing cultures are significantly lower than those generated by amyloidogenic APP processing in differentiated neuronal culture models derived from human FAD iPS cells or cells transduced with FAD genes (Israel and Goldstein, 2011; Choi et al., 2014; Muratore et al., 2014).

#### Neuronal Differentiation

##### Early differentiation

**FIGURE 3:**
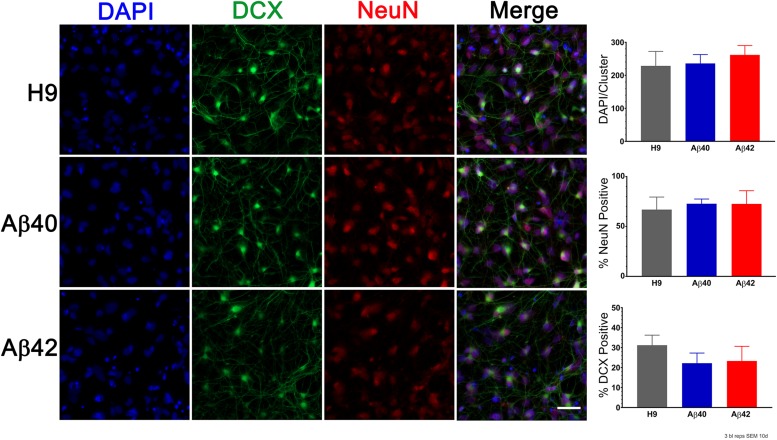
Editing does not significantly affect early stage neuronal differentiation. **(Left)** Representative images of 10-day-old cultures stained with antibodies to DCX (doublecortin, green) to visualize early stage neuronal differentiation, NeuN (red) to visualize more mature neurons and DAPI (blue) to assess total cell number. **(Right)** Quantification of positively stained cells for each marker indicate that there were no genotype-specific differences (ANOVA, Dunnett’s correction). Bars are the mean (SEM) of three biological replicates. Scale bar = 30 μm.

Alzheimer’s disease is a chronic and progressive neurodegenerative disease that only appears later in life. We observed no consistent genotype-specific differences in morphology of ES stage culture, EB formation, or the earliest stages of culture in neuronal differentiation medium (see Supplementary Methods and Data and Supplementary Figure S1). Additionally, earlier stage embryoid bodies (7-day-old) lose their initial positive staining for OCT4 (stem cell marker) and acquire Nestin staining (early neural differentiation marker) at a similar time independent of editing. The appearance of differentiation markers in 10-day-old cultures is shown in Figure 3. The total cell number (DAPI), DCX-positive cells (doublecortin, early stage neuronal differentiation), and NeuN-positive cells were not significantly different among the three genotypes (ANOVA, Dunnett’s correction). We conclude that genomic editing and APP heterozygosity do not appear to affect neurogenesis or early neural development in our cultures and that the majority of cells (60–70%) can be classified as neurons after 10 days of differentiation. Hereafter, all culture ages for differentiated cells are specified relative to EB dissociation and plating taken as day 0.

##### Morphological appearance of differentiated cells

**FIGURE 4:**
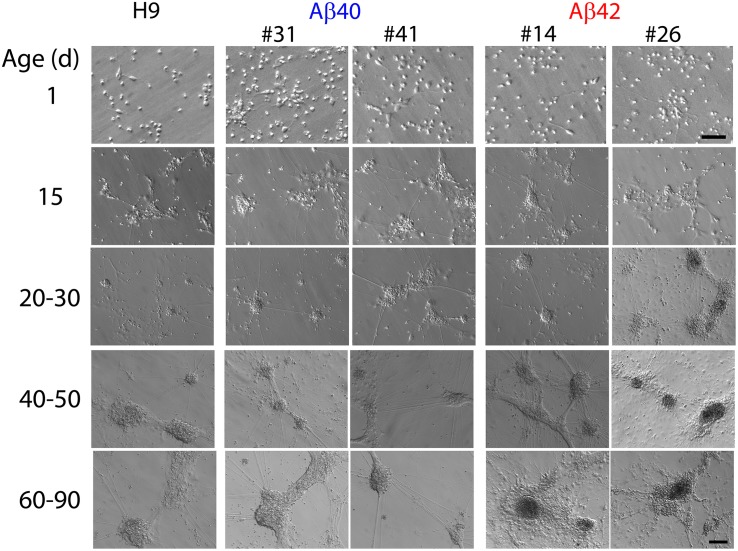
Representative Hoffman interference contrast images of unedited H9 parental cells and two independently isolated clones for each edited genotype (Aβ40:#31, #41 and Aβ42:#14, #26) at different culture ages. Isolated cells in 1 day cultures begin to cluster together a few days after plating. By ∼10–15 days of differentiation all three genotypes form more recognizable neuronal clusters (NCs) which are attached to the culture surface and elaborate neural processes which connect with adjacent NCs. Morphologic appearance of all three genotypes was generally similar up to ∼30–40 days of culture. The absolute size of NCs varied across independent differentiations; however, there were no significant differences among the three genotypes up to ∼30 days of age (ANOVA, Dunnett’s correction). After ∼20–30 days, Aβ42 genotypes begin to exhibit a granular and darker appearance (especially evident in the Aβ42 clone #26 30-day image) and the somal regions are no longer firmly attached to the culture surface but tethered by their neuronal processes. After 50–60 days, essentially all Aβ42 genotypes exhibit this type of morphology as do many of the Aβ40 cultures at culture times greater than ∼70–90 days. We did not observe any consistent clone-specific differences for edited genotypes. Scale bars = 10 μm for 1-day culture and 100 μm for other ages.

Consistent with the neuronal maker data the morphological appearance of all three genotypes, as well as the independently edited clones, remains quite similar up to about 30 days of culture (Figure 4). One-day-old cultures have only isolated cells, a few of which appear to exhibit short processes. By ∼15 days, cells appear to self-organize into loosely defined neural clusters (NCs) and elaborate neural processes, some connecting to adjacent clusters. The size of the NCs increases slightly between 20 and 30 days and begins to appear more three-dimensional. Many NCs are connected to each other by neural processes at this stage. The size of NCs in both edited genotypes often appeared slightly larger compared to H9 cultures, but this was not statistically significant (ANOVA, Dunnett’s corrected) and absent by 40 days.

At culture times of 40–50 days, Aβ42 NCs usually had a more granular appearance and were darker than the other genotypes. In one case we also observed this morphologic change as early as 30 days (see Figure 4, Aβ42 clone #26). This morphologic appearance was more prominent in Aβ42 NCs older than 60 days and thus appears to specific to the Aβ42-edited cells. The neuronal soma for both edited genotypes lost firm attachment to the culture substrate after ∼60–70 days but remained loosely tethered to the culture dish through their neural processes. This could be easily observed when gently moving the culture dish and was not seen in the unedited H9 cultures. Notably, we were not able to culture viable cells for either edited genotype for any time longer than 120 days. In contrast, unedited H9 cultures could be maintained for >266 days.

We conclude that direct Aβ expression in edited cell lines thus decreases the survival time of neurons and results in specific morphologic changes, especially apparent at earlier culture times for Aβ42 edits. The absolute size of NCs had considerable variation in independent differentiations but this was a property of all three genotypes. These morphologic descriptions were generalized from observations made by three different investigators on 15 independent differentiations over a period of >2 years using several different lots of media and supplements and two independently isolated clones for each edited genotype.

#### Aβ Accumulation

##### Intracellular accumulation of Aβ aggregates/oligomers

The main objectives of this study were to document putative AD-related phenotypes resulting from direct Aβ expression in human neurons and compare the rate or extent of phenotypic differences between Aβ40 and Aβ42. The most commonly observed AD-related phenotypes present in most animal models as well as several iPS culture models is the accumulation of aggregated Aβ produced by amyloidogenic APP proteolysis [see Mungenast et al. (2016) and Sasaguri et al. (2017) for reviews].

**FIGURE 5:**
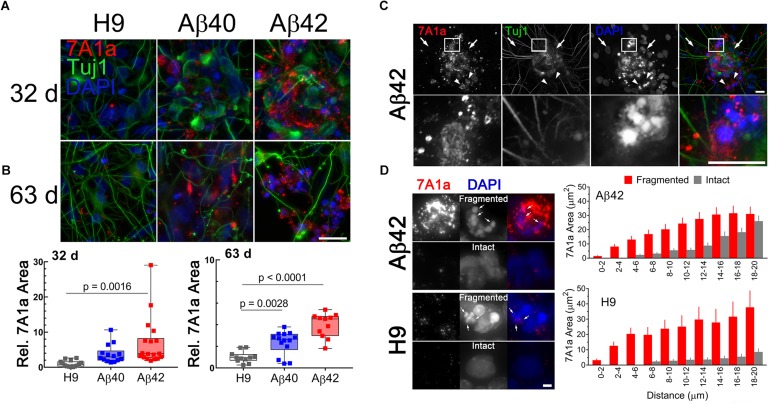
Accumulation of aggregated/oligomeric Aβ is time dependent and more prominent in Aβ42 relative to Aβ40 edited cultures and is associated with pyknotic nuclei, even in unedited H9 samples. **(A)** Maximum intensity *Z*-projections of NCs fluorescently stained with anti-Tuj1 (neuronal, green) and anti-Aβ 7A1a (aggregated/oligomeric Aβ, red) antibodies in 32- or 63-day-old cultures. Consistently, the area of 7A1a positive staining is greater in Aβ42 NCs, intermediate in Aβ40 NCs, and much lower in unedited H9 cultures. Staining is primarily intracellular and initially appears as small puncta which are more obvious in areas of lower staining intensity. **(B)** Box and whisker plot of relative 7A1a staining in individual NCs (normalized to Tuj1 staining). The line in the box is the median value, whiskers are the range. Data is from four independent differentiations. NCs from Aβ42 cultures have significantly greater accumulation of aggregated/oligomeric Aβ at 32-days (ANOVA, Dunnett’s correction) relative to H9. Accumulation of Aβ40 in cultures appears higher than H9 but is not significant at this age. Mean relative accumulation of 7A1a staining ± SEM was: H9 = 1 ± 0.235, Aβ40 = 3.77 ± 0.704, and Aβ42 = 6.93 ± 1.63. In 63-day-old cultures, both Aβ40 and Aβ42 are significantly different relative to H9. The mean relative areas are: H9 = 1 ± 0.157, Aβ40 = 2.34 ± 0.287, and Aβ42 = 3.959 ± 0.337. **(C)** 7A1a staining is present primarily in areas near pyknotic/fragmented DAPI-stained nuclei (i.e., small intensely fluorescent structures, arrowheads) and absent from cells with normal nuclei (i.e., large, weak DAPI fluorescence, arrows). Images are from a single optical section of a 32-day-old Aβ42 sample (top row) with a magnified view (bottom row) of the indicated rectangular area. **(D)** Association of 7A1a and pyknotic nuclei is not dependent on editing; (Left) images of fragmented or intact nuclei from Aβ42 or H9 cultures and (Right) spatial distribution of 7A1a fluorescence relative to the center of mass for DAPI staining. Bars are the mean (SEM) area of 7A1a staining in individual concentric circles centered on the DAPI staining. Data are from at least 60 nuclei or pyknotic nuclei from three independent differentiations of 32-/34-day-old cultures. Scale bar in panels **A** = 10 μm, **C** = 20 μm, and **D** = 4 μm.

We double stained cultures with anti-Aβ antibody (7A1a) which specifically recognizes low and high molecular weight aggregates/oligomers of Aβ40 or Aβ42 (van Helmond et al., 2010; Ling et al., 2014) and anti-Tuj1 (TUBB3 gene product) to confirm neuronal cellular identity. In 32-day-old cultures the level of 7A1a positive staining is genotype specific (Figure 5A). The relative area of 7A1a staining (normalized to Tuj1) was minimal in H9, intermediate in Aβ40, and significantly higher in Aβ42 cultures. Compared to unedited H9 cultures, the area of 7A1a staining was approximately twofold higher in Aβ40 cultures (but not statistically different from H9) and approximately threefold higher in Aβ42 cultures (*p* < 0.0016) at 32 days (Figure 5B). At a later culture age (63 days) the average accumulation of 7A1a positive staining relative to H9 increased to approximately threefold in Aβ40 and ∼4.5-fold in Aβ42 cultures. Accumulation of aggregated/oligomeric Aβ is thus progressive and faster for Aβ42 relative to Aβ40 cultures. This result is consistent with the biophysical aggregation properties of these two peptides *in vitro* (Bharadwaj et al., 2009) and since both edited genes are expressed at comparable levels suggests that Aβ42 may be removed at a slower rate. Both edited genotypes have less Tuj1-positive staining which was especially evident in older Aβ cultures but not in older H9 cultures (5A).

We confirmed that 7A1a staining in Aβ-edited neurons is primarily intracellular by constructing maximum intensity orthogonal projections of image stacks (see Supplementary Methods and Data and Supplementary Figure S2). The staining appears to be localized near pyknotic nuclei, a characteristic of dead or dying cells (i.e., nuclear condensation and fragmentation). Normal neuronal nuclei are large and only weakly stained with DAPI while pyknotic bodies are smaller and have significantly more intense DAPI fluorescence. Figure 5C shows this spatial relationship in a 32-day-old Aβ42 culture. Larger areas of 7A1a staining were generally absent in areas near normal nuclei but common near pyknotic nuclei. Whenever 7A1a staining was occasionally present close to normal nuclei the staining appears to be punctate (possibly vesicular).

We also noticed that a few cells in unedited H9 cultures with 7A1a positive staining also seemed to be near pyknotic nuclei (Figure 5D, left panel). We tested this spatial relationship by placing a counting grid of concentric circles (radius increased in 2 μm increments) over the center of mass for normal pyknotic bodies in H9 and Aβ42 cultures. The area of 7A1a staining in each ring relative to the distance from the center of mass is plotted as a histogram in Figure 5D (right panel). Pyknotic nuclei have more 7A1a staining nearby relative to normal intact nuclei. Surprisingly, this spatial relationship is similar for both Aβ42 edited and unedited H9 neurons. This suggests that pyknosis could be caused by aggregated/oligomeric Aβ derived from either direct expression or through APP amyloidogenic processing.

#### Chronic Progressive ND

**FIGURE 6:**
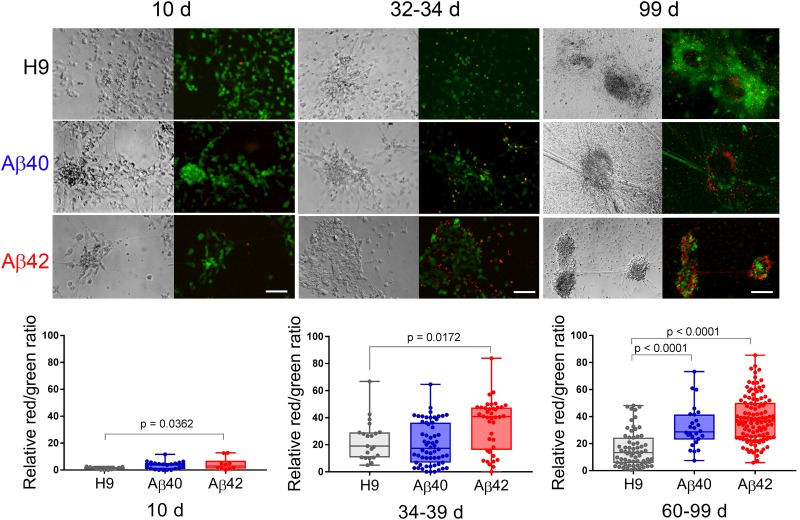
Aβ42 and Aβ40 edited cultures undergo chronic progressive ND. **(Top)** Hoffman extended depth-of-field images **(left)** with a corresponding fluorescent maximum intensity projection **(right)** at three different culture ages. Green fluorescence (calcein-AM) and red fluorescence (ethidium homodimer) was used to estimate live or dead cells. **(Bottom)** Quantitation of relative ratio of dead/live cells. Relative to unedited H9 cultures there are significantly more dead neurons in Aβ42 samples at all three culture ages (ANOVA, Dunnett’s corrected). Aβ40 samples have significantly more dead neurons but only in cultures older than 60 days. Mean values (±SEM) for 10-day-old samples were: H9 = 1.173 ± 0.289, Aβ40 = 3.4 ± 0.643, and Aβ42 = 4.49 ± 1.471; for 34–39-day-old samples: H9 = 23.9 ± 3.226, Aβ40 = 20.79 ± 2.025, and Aβ42 = 35.00 ± 2.974; and for >60-day-old samples: H9 = 16.02 ± 1.612, Aβ40 = 32.36 ± 3.016, and Aβ42 = 38.46 ± 1.588. Each data point represents an individual NC collected from a total of eight individual differentiations. The line inside the box is the median and the whiskers are the range. Scale bar = 100 μm.

Alzheimer’s disease is a chronic progressive disease with end-stage neuronal cell death, a phenotype that has been particularly difficult to document in most current experimental models. We used a fluorescent live/dead assay to assess neuronal viability at three different culture ages. Representative morphological and fluorescent images of the same field are shown in Figure 6 (top). Despite a normal morphologic appearance and similar numbers of neurons in 10-day-old cultures, we found a slightly higher proportion of ethidium homodimer fluorescence (dead cells) in Aβ42 cultures even at this early culture stage (Figure 6, bottom). At an intermediate culture age (34–39 days) when Aβ42 neuronal clusters have significantly fewer synapsin puncta, the relative ethidium homodimer fluorescence was greater in Aβ42 compared to either Aβ40 or H9 cultures. When maintained for longer times (i.e., >∼60 days) both Aβ40 and Aβ42 edited cultures exhibit significantly more relative ethidium homodimer fluorescence compared to unedited H9 cultures. Since most cells under our culture conditions are positively identified as neurons (∼70–90% Tuj1 positive), we conclude that editing results in progressive ND. This phenotype appears at a faster rate for Aβ42 cells relative to Aβ40 cells and is dependent on editing. No viable cells remained in edited culture older than 120 days while H9 cultures still appeared healthy even after 266 days. This edit-specific progressive ND also appears to be chronic because of the extended time necessary for its elaboration.

#### Synaptic Density

**FIGURE 7:**
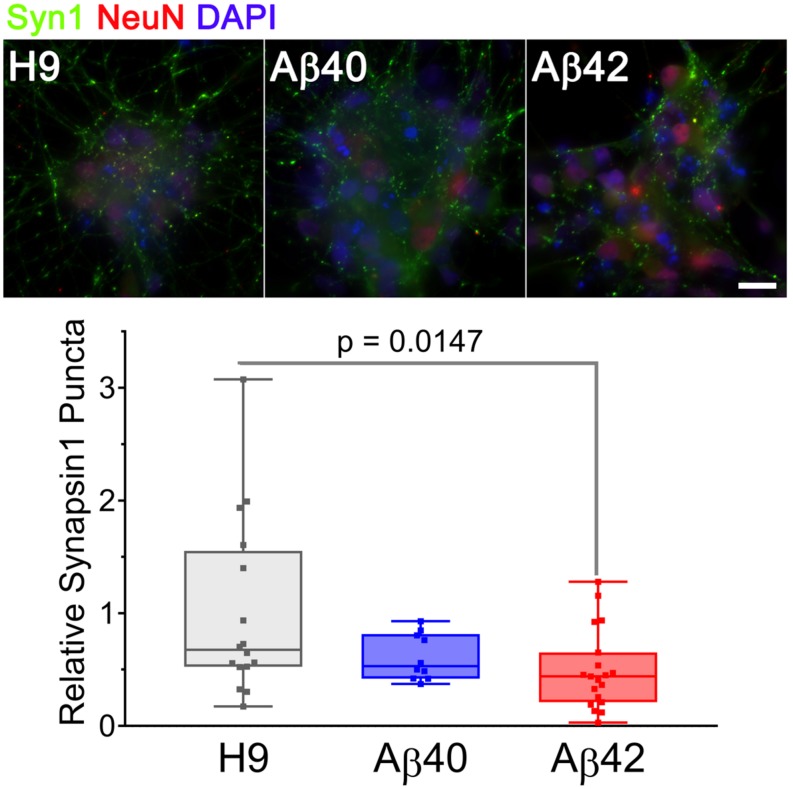
Aβ42 edited NCs have fewer synapsin1-stained puncta in 34-day-old cultures. Images are maximum intensity projections of three adjacent 0.05 mm spaced optical sections stained with anti-synapsin1 (synaptic marker, green) and anti-NeuN (mature neurons, red) antibodies and DAPI (total cells, blue). Synapsin1 positive puncta were counted in individual NCs from three different differentiations normalized DAPI and analyzed (ANOVA, Dunnett’s corrected). The number of synapsin1 puncta was significantly less for Aβ42 cultures. The relative number of puncta ± SEM was: H9 = 1 ± 0.198, Aβ40 = 0.618 ± 0.065, and Aβ42 = 0.492 ± 0.081. Data were from three independent differentiations. Scale bar = 20 μm.

A decrement in the number of synapses is a consistent and early AD phenotype that correlates well with cognitive decline, even during preclinical disease stages (Forner et al., 2017). Several transgenic mouse models exhibit synaptic deficits, but we are unaware of this phenotype being described in human AD cell culture models. We stained 34-day-old cultures with anti-synapsin 1 antibody (a presynaptic marker) to estimate the number of synapses present in neuronal clusters from the different genotypes. As shown in Figure 7, all three genotypes at this culture stage have a significant number of synapsin positive puncta. There are, however, ∼50% fewer synapsin positive puncta in Aβ42-edited samples (*p* < 0.0147) relative to unedited H9 samples. Aβ40 samples had ∼20% fewer synapsin puncta but did not reach significance. There is thus a graded genotype dependent difference in the number of synapsin puncta at this culture stage: H9 > Aβ40 >> Aβ42. Additional work will be necessary to distinguish if the Aβ42 synaptic deficiency was due to decreased synaptogenesis or increased synaptic loss. Our results establish that synaptic number is reduced to a greater extent in Aβ42 compared to Aβ40 cultures, a result that is consistent with the concept that Aβ negatively affects synaptic capacity (Forner et al., 2017).

#### Endolysosomal Pathway Phenotypes

Dysfunction of the endolysosomal pathway plays an important role in several neurodegenerative diseases, including AD (Nixon, 2017). Pathway dysfunction is a consistent feature of several animal and cellular AD models (Israel et al., 2012; Nilsson et al., 2013) as well as an early phenotype in AD (Cataldo et al., 2004) and can be inferred by accumulation of an abnormal number or size of characteristic vesicles.

**FIGURE 8:**
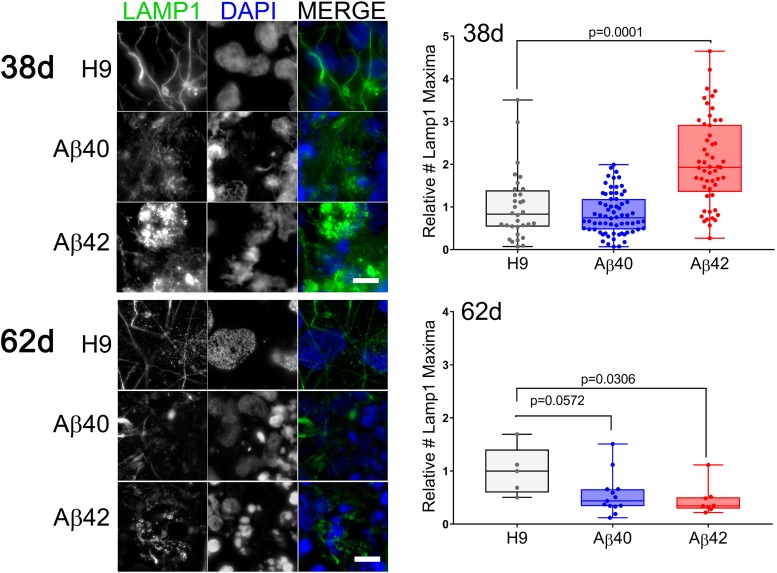
The number of LAMP1-positive vesicles is affected by editing. **(Left)** Fluorescence maximum intensity projections of two adjacent optical sections stained with anti-LAMP1 antibody (green) or DAPI (blue) at two different culture times. **(Right)** The relative number of LAMP1-positive puncta (normalized to DAPI) in individual NCs was greater in 38-day-old Aβ42 samples relative to H9. In 62-day cultures both Aβ42 and Aβ40 (not significant) samples have fewer LAMP1 objects relative to H9 (ANOVA, Dunnett’s corrected). Data are from three independent differentiations of 38-day cultures and two independent differentiations of 62-day cultures. Mean values (±SEM) for 38-day samples were: H9 = 1 ± 0.139, Aβ40 = 0.84 ± 0.059, and Aβ42 = 2.064 ± 0.142; and for 62-day samples: H9 = 1 ± 0.205, Aβ40 = 0.553 ± 0.106, and Aβ42 = 0.453 ± 0.101. The line inside the box is the median and the whiskers are the range. Scale bar = 10 μm.

**FIGURE 9:**
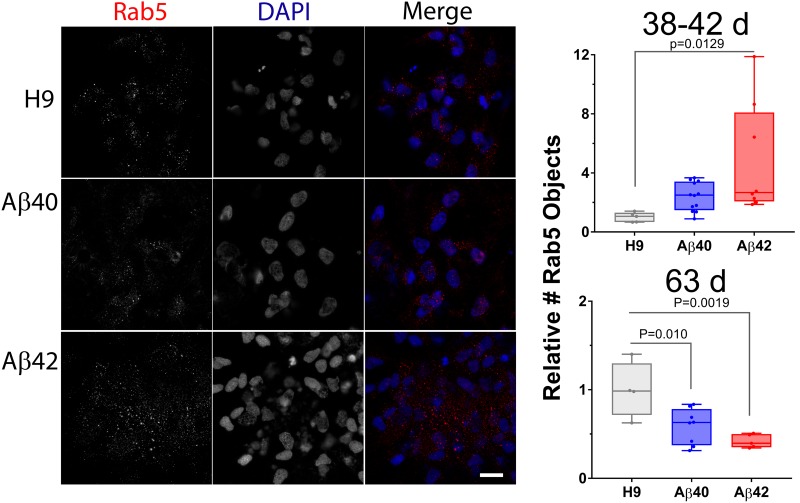
The number of other endolysosomal vesicles is affected by editing. Rab5-positive objects in NCs is greater in 38–42-day-old Aβ42 samples. At a later culture age (63 days) both Aβ40 and Aβ42 samples have fewer LAMP1 objects. **(Left)** Maximum intensity projections of two adjacent optical sections (1 μm spacing) stained with anti-Rab5 antibody (early endosome marker, red) and DAPI (blue). **(Right)** The relative number of Rab5 puncta (normalized to DAPI) is greater in 38–42-day-old cultures for Aβ42 edited samples and less for both Aβ42 and Aβ40 edited samples in 63-day cultures (ANOVA, Dunnett’s corrected). Data are from individual NCs from three independent differentiations for 38–42-day-old cultures and two independent differentiations for 63-day cultures. Images for 63-day-old cultures can be found in Supplementary Figure S8. Mean values (±SEM) for 38–42-day-old samples are: H9 = 1 ± 0.1448, Aβ40 = 2.39 ± 0.2767, and Aβ42 = 4.80 ± 1.333; and for 63-day samples are: H9 = 1 ± 0.1584, Aβ40 = 0.586 ± 0.071, and Aβ42 = 0.4198 ± 0.0341. Scale bar = 10 μm

Using vesicle type-specific antibody staining we counted the relative number of punctate vesicular structures in neurons. Figure 8 presents representative images and quantitative analysis for 38- and 62-day-old cultures stained with anti-lysosomal associated membrane protein 1 (LAMP1) antibody. There was an approximately twofold increase in LAMP1-positive puncta in 38-day-old Aβ42 cultures relative to either Aβ40 or unedited H9 cultures. This finding agrees with the reduced neuronal viability in Aβ42 samples and synapsin1 puncta at this culture stage. In older cultures (62 days), the number of Lamp1 puncta relative to unedited H9 cells was decreased ∼60% in Aβ42 and ∼50% in Aβ40 samples (although not significant). This decrease thus correlates with ND present in both edited genotypes in older cultures. Abnormal accumulation of lysosomal-related vesicles may thus be a consequence of direct Aβ expression in human neurons. The number of Rab5-stained puncta, a marker for early endosomes necessary for vesicular maturation leading to lysosomal fusion (Poteryaev et al., 2010) is shown in Figure 9. The pattern is similar to LAMP1 puncta. There was a significant increase in Rab5 puncta in 38–42-day-old Aβ42 relative to H9 samples and a non-significant increase in Aβ40 samples. Both edited genotypes also showed a significant decrease in Rab5 puncta in older 63-day cultures.

We also obtained object counts for Rab3A staining, a synaptic vesicular gene important for regulating normal synaptic neurotransmission (Schluter, 2004) and LC3B staining, an autophagosome vesicle marker necessary for delivering mature autophagic/endosomal vesicles to lysosomes for cargo digestion which has been associated with AD (Boland et al., 2008). The number of Rab3A puncta were not significantly different among any of the genotypes in 43-day-old cultures but both genotypes exhibit a reduction in 63-day-old cultures. Both Aβ40 and Aβ42 samples had a reduction in LC3B puncta in 43-day-old cultures (only Aβ40 was significant) as well as in 63-day cultures. Results were more variable for all three genotypes and counts are presented in Supplementary Methods and Data and Supplementary Figure S3.

These results indicate that endolysosomal pathway dysfunction is associated with Aβ-edited samples and that Aβ42 samples appear to be affected at earlier times and to a greater extent than Aβ40 samples. These changes were not likely attributable to changes in gene expression for key vesicular genes since qRT-PCR analysis did not find any genotype-specific expression differences (see Supplementary Methods and Data and Supplementary Figure S4). Since we are directly expressing Aβ in edited cultures, these potential AD-related phenotypes are likely to be independent of APP amyloidogenic processing believed to occur in large part within endolysosomal vesicles (Haass et al., 2012).

##### Aβ-dependent differential gene expression

The edited cell lines present a particularly favorable opportunity for whole transcriptome RNA-Seq analysis to identify differentially expressed genes (DEGs) that may be mechanistically linked to Aβ-dependent ND. They are not confounded by uncontrolled amyloidogenic APP proteolysis, overexpression of non-Aβ fragments, and are near isogenic. We performed RNA-Seq expression using mRNA isolated from 36- or 38-day-old cultures. This is a stage where phenotypes are either exclusive (i.e., reduced number of synapses, reduced neuronal viability, and increased accumulation of lysosomes and endosomes) or more penetrant (greater accumulation of aggregated Aβ) for the Aβ42 editing compared to Aβ40 editing. RNA isolated from three independent H9 culture samples served as the reference control to identify DEGs for each edited genotype. All three genotypes are heterozygous for the major SAD risk allele (i.e., e4/e3) and thus in an appropriate human genetic context relevant to a large proportion of SAD cases (Corder et al., 1993).

**FIGURE 10:**
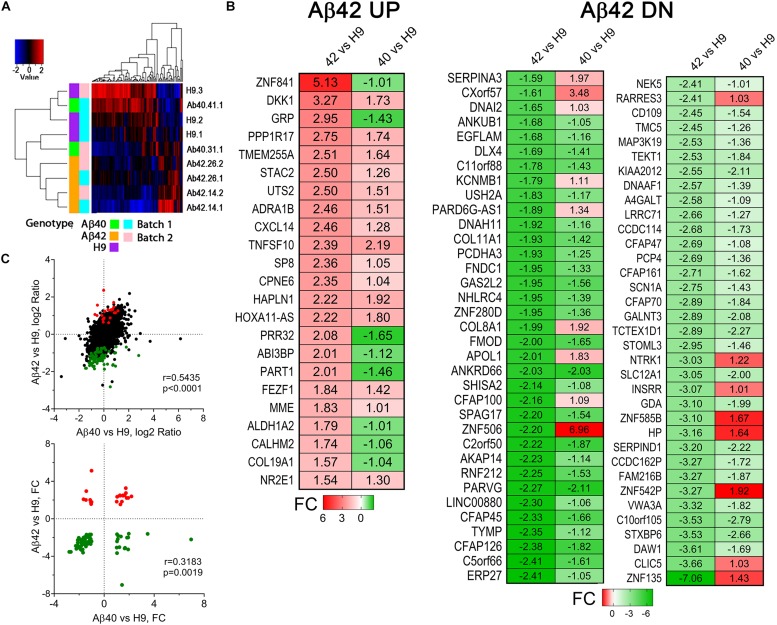
Differentially expressed genes in 34-day-old cultures. **(A)** Cluster analysis of differentially expressed genes (Pearson dissimilarity metric). Aβ42 samples cluster together while Aβ40 and H9 samples overlap. **(B)** Heat map of significant DEGs from RNA-Seq analysis of Aβ42 vs. H9 comparison and Aβ40 vs. H9 comparison. Up (UP) regulated genes (red) and down (DN) regulated genes (green) were sorted by the magnitude of the indicated fold change (FC) values for the Aβ42 vs. H9 comparison. There is a general correspondence in the directional FC values with a relatively larger FC in the Aβ42 samples. **(C)** Pearson correlation confirms significant co-variation of the log2 ratios of all genes (top, significant DEGS in color) as well as the FC values for significant UP and DN regulated genes (bottom).

We tested differential expression for 18,259 genes (i.e., genes that had an FPKM > 0.1 in 50% of samples). Results of hierarchical clustering along with an expression heat-map for the batch centered sample medians of individual samples are shown in Figure 10A. The four Aβ42 samples cluster together on the same branch of the dendrogram. One Aβ40 sample (#31.1) clusters adjacent to the Aβ42 group while the other (#41.1) appears more like unedited H9 samples indicating that whole transcriptome expression is more similar among individual Aβ42-edited samples relative to either Aβ40 or unedited H9 samples which agrees with phenotypic penetrance at this culture age. DEGs may thus be mechanistically associated with Aβ42-dependent affected pathways related to these phenotypes.

We defined DEGs by first identifying genes that vary between Aβ42 vs. H9 and then filtering genes with a similar directional change for Aβ40 that using a more liberal criteria (to avoid keeping genes marginally not significant in the Aβ40 vs. H9 comparison) (see the Supplementary Methods and Data for full details). All 93 DEGs for the Aβ42 vs. H9 comparison are shown in Figure 10B as a fold change (FC) heat map. There were 23 UP and 70 DN (down) regulated genes which were used for functional/annotation enrichment analysis. This number is rather small compared to numerous other AD-related studies of DEGs in patient samples or even iPS cell lines where thousands of DEGs are often identified (Blalock et al., 2004; Israel et al., 2012; Zhang et al., 2013; Annese et al., 2018). Note that the directional FC was similar for most genes in the Aβ40 samples compared to Aβ42. The Pearson correlation coefficients for log_2_ ratios of all Aβ42 vs. H9 compared to Aβ40 vs. H9 genes were 0.5434 (all genes, linear-regression *p*-value < 0.0001, Figure 10C, top), and the correlation coefficient for the DEG FC values is 0.3183 (DEGs, linear regression *p*-value = 0.0019, Figure 10C, bottom). Aβ-dependent changes in gene expression thus appear similar for Aβ42 and Aβ40 samples. A complete list of all detected genes, the genotype-specific average log2 RPKM values, log2 ratios of the H9 DEG comparisons, FC values, statistics, and DEG status is included in Supplementary Table S1.

Gene Ontology enrichment analysis of the UP and DN regulated genes for the Aβ42 vs. H9 comparison did not identify functional enrichment for UP genes after correcting for false discovery rate (FDR). The statistical power of this approach, however, is likely limited when using a small number (23) of input DEGs. For DN genes, however, 13 out of 70 (19%) were related to cilia functions and were significantly overrepresented (i.e., FDR < 0.05) (CCDC114, CFAP100, CFAP126, CFAP45, CFAP70, DAW1, DNAAF1, DNAH11, DNAI2, SPAG17, STOML3, TEKT1, USH2A). Interestingly, five of these “cilia” genes (DAW1, DNAH11, DNAI2, GDA, and TEKT1) were also differentially expressed in a hippocampal AD vs. non-AD RNA-Seq study (Annese et al., 2018) suggesting that cilia-related pathways may also be affected in AD. Using unadjusted *p*-values, microtubule and cytoskeletal genes were also over represented (CCDC114, CFAP100, CFAP126, CLIC5, DNAAF1, DNAH11, DNAI2, GAS2L2, PARVG, SPAG17, TEKT1, USH2A) as well as genes associated with vesicle lumen (COL11A1, COL8A1, ERP27). Overrepresented molecular functions included neurotrophin receptor associated terms (NTRK1) and peptidase regulatory roles (CD109, SERPINA3, SERPIND1). The complete GO results are included in Supplementary Table S2.

We also used GATHER^2^ to broaden the search for relationships/pathways in the Aβ42 DEGs. Two GO terms were statistically significant for UP genes (FDR < 0.05): *GO:0007267*: cell–cell signaling (ADRA1B, CPNE6, CXCL14, MME, TNFSF10, UTS2) and *GO:0007154*: cell communication (ADRA1B, COL19A1, CPNE6, CXCL14, DKK1, GRP, HAPLN1, MME, STAC2, TNFSF10, UTS2). DN genes included two overlapping GO terms: GO:0015698: inorganic anion transport and GO:0006820: anion transport (CLIC5, COL11A1, COL8A1, SLC12A1). KEGG pathways with an FDR < 0.25 included hsa04080: Neuroactive ligand–receptor interaction (ADRA1B, GRP, UTS2) and hsa05010: AD [MME] for UP genes. DN genes were hsa04512: ECM–receptor interaction (COK11A1, FNDC1). Complete GATHER results are included in Supplementary Table S3.

gene set enrichment analysis (GSEA) KEGG analysis^3^ is an additional way to discover potential pathway relationships and is not limited by using a small list of input genes since input can be a rank order list of FC values for all detected genes. We performed GSEA using a rank ordered FC list (18,233 genes) and compared these to all KEGG pathways. The Aβ42 vs. H9 list identified 118/170 KEGG gene sets that were upregulated. Twenty-two had a nominal *p*-value < 0.05 and three of these had an FDR < 25%. The top scoring KEGG pathway was NEUROACTIVE LIGAND RECEPTOR INTERACTION (hsa04080, Normalized Enrichment Score = 2.12, *p* < 0.01, FDR = 0.014). Our gene list included 79 of the 219 (36%) genes in this pathway suggesting widespread changes in neuroactive ligand receptor signaling was a consequence of direct Aβ42 expression. This can plausibly be related to the DN regulated expression of “cilia”-related genes since primary cilia in neurons are believed to be a major organelle signaling hub known to express a host of neuroactive ligand receptors (Guemez-Gamboa et al., 2014). No KEGG pathways reached significance (FDR < 0.05) for DN genes or for a separate analysis of ranked Aβ40 vs. H9 DEG FC values. Summary results for the top 20 GSEA KEGG pathways for Aβ42 vs. H9 genes along with details of the KEGG NEUROACTIVE LIGAND RECEPTOR INTERACTION pathway are included in Supplementary Table S4.

**FIGURE 11:**
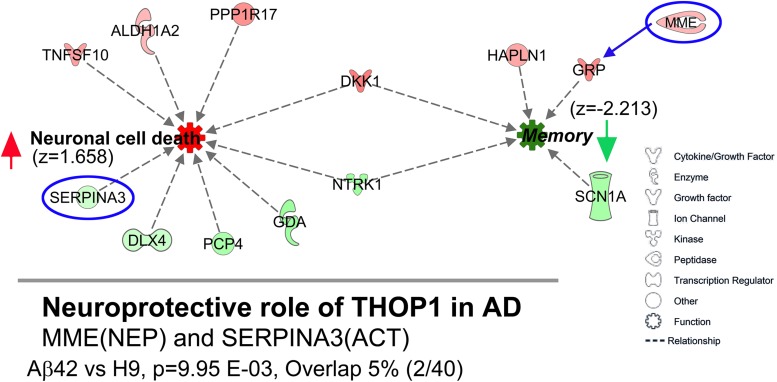
DEGs are potentially related to Alzheimer’s relevant pathways and functions. **(Top)** IPA pathway analysis of DEGs in the Aβ42 vs. H9 comparison identified “decreased memory” (*z* = –2.213) and “increased neuronal cell death” (*z* = 1.658) as the lowest and highest scoring functional pathways. Individual genes are shown as graphic symbols representing molecule type and color coded by FC values (red = UP, green = DN). **(Bottom)** The most relevant IPA disease-related canonical pathway was “Neuroprotective role of THOP1 in Alzheimer’s disease” (*p* = 9.95*E*–03, overlap = 2 of 40 total genes in this pathway). The pathway genes were MME (aka NEP, neprilysin) an Aβ degrading metalloproteinase and SERPINA3 (aka ACT, alpha-1 antitrypsin) a protease inhibitor found in AD plaques (**Top**, circled in blue). MME is not directly included in the IPA “decreased memory” function but could potentially be related through its relationship to GRP.

We also analyzed DEGs with Ingenuity Pathway Analysis (IPA). Remarkably, for the Aβ42 vs. H9 comparison, the highest and lowest *z*-scores were obtained for the functions “Increased Neuronal Cell Death” (*z* = 1.658) and “Decreased Memory” (*z* = −2.213), two biological processes with obvious relevance to AD. Figure 11 shows the individual DEGs identified by this analysis color coded by intensity for FC values. The “Decreased Memory” and “Increased Neuronal Cell Death” pathways are connected through the overlap of DKK1 and NTRK1. IPA disease-related pathways returned the “Neuroprotective Role of THOP1 in AD” as the top scoring canonical pathway (*p* = 9.95*E*−3). This pathway was also significant for a hippocampal DEG analysis of LOAD RNA-Seq data (Annese et al., 2018). Thimet oligopeptidase (product of THOP1) is reportedly neuroprotective for Aβ toxicity in cortical neurons and can degrade soluble Aβ but not aggregated Aβ42 (Yamin et al., 1999; Pollio et al., 2008). The DEGs in the Aβ42 vs. H9 comparison represent only a small fraction of the 40 genes in this pathway. They were MME (aka NEP, neprilysin) and SERPINA3 (aka ACT) (indicated on the bottom of Figure 11). MME is not directly related to decreased memory in IPA, but is included because of its potential indirect relationship through GRP (Saghatelian et al., 2004). SERPINA3 is a member gene of the “Neuronal Cell Death” category in IPA and both genes are part of the extracellular arm of the THOP1 in AD pathway in IPA. MME is an Aβ degrading enzyme with increased expression in the Aβ42-edited cells and SERPINA3 is a serine protease inhibitor with decreased expression which co-localizes with Aβ in AD plaques (Abraham et al., 1988).

There is no general consensus regarding a “signature” set of AD-related DEGs, especially those that related to early LOAD pathogenic mechanisms making it challenging to compare our expression data with patient samples that likely contain signals from many different non-neuronal cell types, co-morbidities, and many complex combinations of genetic variance. Nevertheless, we did find some potentially relevant comparisons. For example, the GeneCards database^4^ has 6,672 genes identified as “Alzheimer’s-related genes.” This is a rather large list not restricted to DEG analysis but also including GWAS hits as well as other types of associations. For our UP genes we found 10/23 (43%) that overlapped (TNFSF10, DKK1, GRP, CALHM2, MME, ALDH1A2, CXCL14, PPP1R17, TMEM255A, HAPLN1) and 17/70 (24%) DN genes (SHISA2, DNAH11, SERPIND1, SCN1A, APOL1, HP, ERP27, SERPINA3, STXBP6, CFAP70, PARVG, GDA, PCP4, NTRK1, TMC5, STOML3, RARRES3) suggesting some potential AD relevance. A recent RNA-Seq analysis of hippocampal tissue from SAD vs. non-SAD patient samples (Annese et al., 2018) identified 2,064 DEGs. We found only 3 out of 23 (13%) UP genes overlapped (not statistically significant; Fisher’s exact test, *p* = 0.46) (HAPLN1, CPNE6, TNFSF10). In contrast, 22 out of 70 (31%) DN genes overlapped (Fisher’s exact test, *p* = 2.5 × 10^–6^) (DAW1, FAM216B, GDA, TCTEX1D1, PCP4, CCDC114, LRRC71, A4GALT, MAP3K19, TEKT1, CD109, TMC5, RARRES3, LINC00880, PARVG, ANKRD66, FNDC1, DNAH11, C11orf88, ANKUB1, DNAI2, SERPINA3). Four of these DN genes had an opposite directional FC, while others agreed with our DEGs. This significant overlap suggests that DEGs in our Aβ-dependent neuronal model may thus have relevance to the AD, including the possible involvement of cilia dysfunction as mentioned above.

## Discussion

Many genes, molecules, cell types, and pathways have been implicated in AD. This wealth of information comes from numerous patient observation or investigation of a variety of experimental AD models but has not yet been developed into effective treatments. The three interrelated factors which may account for the clinical failures are disease complexity; limited experimental accessibility of patient material; and phenotypic deficiencies or artifacts in all available AD models (Ashe and Zahs, 2010; Bales, 2012; Sasaguri et al., 2017). In this study, we reduced the complexity of amyloidogenic APP processing by directly expressing genetic constructs coding for secretory forms of either Aβ40 or Aβ42. Other AD models have also used a direct expression approach (LaFerla et al., 1995; Lewis et al., 2001; McGowan et al., 2005; Iijima-Ando and Iijima, 2009; Abramowski et al., 2012), but ours differ in several important ways. First, we used genomic editing of the *App* gene rather than Aβ transgene over-expression. Direct Aβ expression is thus under control of the normal *App* regulatory DNA and should be expressed at normal levels when and where *App* is normally expressed. Over-expression artifacts are not likely to explain our phenotypic results. Some of the most widely used mouse transgenic AD models have phenotypes that seem related to this common problem (Saito et al., 2014; Sasaguri et al., 2017). Second, we compare edit-specific phenotypes to unedited isogenic parental cells. Third, we use human neurons which likely better capture interactions and processes related to AD.

Alzheimer’s disease has many complex genetic associations. The only difference in edited genotypes compared to unedited parental cells in our model is homologous recombination of the editing cassette in the first intron of *App* which results in *App* heterozygosity. *App* heterozygosity, however, is unlikely to directly explain our phenotypic results since independently generated Aβ40 or Aβ42 lines are both heterozygous, yet they display significant differences in phenotypic initiation and rate of progression. Early developmental functions of APP have been described (Freude et al., 2011), but both edited genotypes developed and differentiated into neurons essentially identical to unedited parental cells. Additionally, APP mRNA levels in edited lines, while consistent with heterozygosity, maintained the same relative distribution of several different qRT-PCR amplicons compared to unedited samples suggesting that editing does not significantly affect the pattern of APP mRNA processing of the unedited allele. Homozygous deletion of *App* in mice also has limited phenotypic consequence (Zheng and Koo, 2006; Müller and Zheng, 2013) while heterozygous deletions were indistinguishable from wild-type littermates (Zheng et al., 1995). Some work suggests that non-Aβ fragments may contribute to AD-like phenotypes (Zheng and Koo, 2006; Müller and Zheng, 2013). While we have not directly studied this, our direct Aβ expression strategy makes it unlikely. Finally, we made parallel observations on at least two independently isolated clones for each edited genotype and phenotypic results were similar suggesting that they are not due to any off target genetic changes. We thus conclude that all edit-specific phenotypes can be attributed to direct expression of either Aβ40 or Aβ42.

An overwhelming body of evidence has been formalized into the dominant “Amyloid Hypothesis” of AD which suggests that structurally imprecise Aβ oligomers are responsible for initiating a cascade of downstream progressive pathology terminating in ND (Selkoe and Hardy, 2016). Nevertheless, unresolved conflicts regarding the pathogenic significance of Aβ to AD remain (Benilova et al., 2012; Musiek and Holtzman, 2015; Selkoe and Hardy, 2016). Oligomer formation is complex and incompletely defined, especially with respect to demonstrations of neuronal toxicity (Walsh et al., 2000; Benilova et al., 2012; Teplow, 2013). The mechanistic details linking Aβ oligomers to downstream molecular and cellular processes are also not entirely clear. Part of the reason for these conflicts may be related to the absence of AD human culture models (Mungenast et al., 2016; Arber et al., 2017) or amyloidogenic rodent models (Sasaguri et al., 2017) that have progressive ND phenotypes. One principle finding of our study is that direct Aβ expression appears sufficient to produce chronic progressive ND of cultured human neurons. Oligomeric/aggregated Aβ accumulates intracellularly in differentiated neurons and precedes the appearance of several other AD-like phenotypes. Amyloidogenic Aβ production is thus not strictly necessary for AD-like phenotypic development. Minimizing amyloidogenic APP processing, however, raises issues of direct relevance to AD. A concern not unique to our model but an issue shared with all current AD models which all have a range of AD associated phenotypic deficiencies.

### Chromic Progressive ND

Stem cell maintenance, EB formation, and neurogenesis were not affected by editing. Only later stage differentiated neurons were subject to edit-specific cell death. The culture age when reduced neuronal viability was first detected was significantly earlier for Aβ42 and progressed at a faster rate compared to the Aβ40. We never observed live neurons in edited cultures older than ∼120 days while parental cultures appeared normal and remained healthy up to 266 days. Taken together these results indicate that direct expression of either Aβ40 or Aβ42 has little effect on undifferentiated stem cells or neuronal early development or neuronal differentiation but rather initiates a month’s long process of chronic progressive ND in the absence of high level amyloidogenic APP processing.

In contrast to our results, similar invertebrate and mammalian models (LaFerla et al., 1995; Lewis et al., 2001; McGowan et al., 2005; Iijima-Ando and Iijima, 2009; Abramowski et al., 2012) all exhibit prominent ND for Aβ42 but not Aβ40 direct expression. Perhaps some human-specific factor could explain this difference. One possibility is that formation of toxic Aβ oligomer/aggregate structures differs in the specific genetic context of the model. For example, oligomer formation of different mixtures of Aβ40 and Aβ42 is dependent on their relative concentrations and further suggested to require an Aβ42 “seed” (McGowan et al., 2005; Kim et al., 2007). *Drosophila* do not contain any Aβ homologous sequence in the *Appl* gene (the fly APP homolog) thus cannot produce Aβ42 via amyloidogenic processing. The absence of Aβ40 ND may reflect the absence of Aβ42. Mammalian direct expression models, however, all produce rodent APP from an endogenous gene which is proteolytically processed into Aβ40 and Aβ42 via the amyloidogenic pathway. The murine Aβ sequences, however, differ by three amino acids relative to the directly expressed human Aβ transgenes. Perhaps unknown mixtures of rodent/human Aβ interfere with formation of neurotoxic oligomers and prevent Aβ40-dependent ND. In our cultures, both directly expressed and amyloidogenic Aβ would have an identical human sequence. An Aβ42-dependent seeding mechanism could thus explain Aβ40 toxicity since we routinely observed small amounts of amyloidogenic Aβ oligomer in unedited cells and this also likely occurs in edited cells which all contain an unedited *App* allele. It is also conceivable that an Aβ42 seeding mechanism could relate to the slower phenotypic elaboration Aβ40 edited neurons relative to Aβ42 since both direct expression and amyloidogenesis could provide an appropriate seed. Additional edited cell lines could be generated in the future to directly address these possibilities.

### Intracellular Aβ Accumulation

Direct expression of Aβ40 or Aβ42 in human neurons resulted in an intracellular accumulation of oligomeric/aggregated peptides detected by 7A1a antibody staining, even though both editing constructs included a normal secretory pathway routing sequence. Coupled with our inability to detect secreted Aβ using ELISA, this indicates that direct expression preferentially retains or rapidly reinternalizes Aβ. This differs from other human AD culture models that produce Aβ via amyloidogenic APP processing. High levels of Aβ accumulate extracellularly in culture media suggesting that Aβ is mostly secreted (Mungenast et al., 2016; Arber et al., 2017). Using either immunocytochemistry or ELISA, only relatively low levels of intracellular Aβ accumulate in these other culture models (Shi et al., 2012; Kondo et al., 2013; Kim et al., 2015; Raja et al., 2016). When we stained cultures using a widely used antibody that recognizes primarily monomeric Aβ (6E19), intracellular staining was diffuse and week, however, staining was more prominent for H9 cells relative to Aβ-edited cells (see Supplementary Methods and Data and Supplementary Figure S5). The method of Aβ production may thus affect its eventual localization. Extracellular plaque-like Aβ aggregates have also been demonstrated in amyloidogenic culture models that use three-dimensional culture support or organoid differentiation. These approaches likely restrict diffusion of secreted Aβ allowing extracellular aggregates to form (Kim et al., 2015; Raja et al., 2016). We were also unable to detect intracellular Aβ protein using ELISA of cell extracts, a finding also observed in some other amyloidogenic human culture models (Israel et al., 2012; Muratore et al., 2017). Low levels of edit-specific transcripts measured in our cells suggest that Aβ protein levels may also be quite low and possibly explain this negative result.

Intracellular Aβ oligomer accumulation correlates best with the rate of ND in our cultures but is this directly relevant to neuronal cell death in AD? Amyloidogenic APP processing occurs at the plasma membrane, where Aβ is released into extracellular spaces where it aggregates into plaques that maintain a dynamic equilibrium serving as an extracellular oligomer source (Haass and Selkoe, 2007). Intracellular vesicle compartments also produce amyloidogenic Aβ which could be preferentially retained (Haass and Selkoe, 2007). Secretion and reuptake of Aβ has also been demonstrated in cultured neurons or early stage AD patient samples (Hu et al., 2009). In other direct expression AD models Aβ42 also appears to be preferentially retained or endocytosed by neurons relative to Aβ40 (Abramowski et al., 2012; Ling et al., 2014). Vesicular Aβ42 in *Drosophila* neurons following direct Aβ expression is primarily oligomeric and accumulates in vesicles with a range of endocytic, autophagic, and lysosomal markers (Ling et al., 2009, 2014). The vesicles are acidic, a condition that would favor formation of oligomers/aggregates.

The relative rates of Aβ production for these two APP processing sites as well as any differential pathogenic significance in AD are not clear (Haass et al., 2012). A recent study of familial AD PSEN mutations associated them with preferential amyloidogenic intracellular Aβ42 accumulation in vesicles suggesting that this Aβ pool may have pathological significance (Sannerud et al., 2016). Other experimental evidence, primarily from animal models also suggest that intracellular Aβ may be more relevant to neuronal pathology (Yang et al., 1995; Oddo et al., 2006; Laferla et al., 2007). Resistance to a broader acceptance of intracellular oligomer toxicity derives from technical deficiencies in establishing its existence in AD patient material. A recent study using well-preserved post mortem human brain tissue, however, has definitively demonstrated intraneuronal Aβ, its age-related accumulation, its oligomeric nature, and an early temporal relationship to other AD phenotypes (Welikovitch et al., 2018). Other human patient-derived AD culture models also have amyloidogenic endolysosomal vesicle phenotypes (Israel et al., 2012). Finally, lysosomal/endosomal/autophagosome dyshomeostasis has long been recognized as an early pathological feature of AD associated with Aβ accumulation (Cataldo et al., 2000, 2004). Our results thus add additional support for an intracellular view of toxic oligomeric Aβ accumulation in human neurons and suggest possible relevance to AD. Of course, both extracellular and intracellular pools of Aβ are dynamically related in AD (Oddo et al., 2006), something that is not modeled in our system.

7A1a antibody has not been widely used in AD studies, possibly because of a report that it might cross react with tropomyosin. Our results don’t support this possibility since tropomyosin mRNA is expressed at similarly high levels for all three genotypes (RNAseq data), but large amounts of 7A1a positive staining were only observed in edited lines in a progressive manner while staining of unedited parental neurons remained very low and was not progressive. In a *Drosophila* direct expression model, 7A1a staining specifically recognizes Aβ oligomer/aggregates in several types of endolysosomal vesicle compartments (autophagosomes, endosomes, and lysosomes) identified by double staining with vesicle marker-specific antibodies (Ling et al., 2009, 2014).

### Progressive ND

With increasing culture times 7A1a staining areas progressively increased in both edited genotypes, remained intracellular, and appeared to be spatially correlated with pyknotic nuclei. However, Aβ42 cultures always had an earlier appearance of 7A1a staining and the area or positive staining expanded at a faster rate relative to Aβ40 cultures. This difference correlates well with the different rates of ND we observed in Aβ42- and Aβ40-edited cultures: dead neurons in Aβ42 cultures appeared sooner and accumulated at a faster rate relative to Aβ40 cultures. Since both edited genotypes had equivalent edit-specific transcript expression the differential 7A1a staining could be a result of an increased ability of Aβ42 to form oligomers/aggregates or faster Aβ40 removal. Both possibilities are consistent with the differential biochemical properties of these peptides. Pyknosis has been associated with AD disease, and is considered a maker for both type 1 and 2 cell death pathways (Yuan et al., 2003; Nixon and Yang, 2012; Ghavami et al., 2014). Our results thus suggest that perinuclear intracellular accumulation of oligomeric/aggregated Aβ could be a cause of neuronal cell death. Interestingly, the spatial relationship of 7A1a staining and pyknosis was not strictly dependent on direct Aβ expression but was also observed in unedited control neurons presumably producing Aβ via amyloidogenic APP processing. Unedited cultures did not show progressive 7A1a staining and had only limited ND relative to Aβ-edited cultures suggesting a mechanism that can limit widespread damage sufficient to prevent ND. Possibly unedited cells accumulate only low levels of oligomeric Aβ or alternatively the Aβ oligomers may be formed in different vesicle compartments following amyloidogenic or direct Aβ production. This latter possibility could be established with additional experiments co-localizing oligomeric Aβ with specific vesicle markers. The specific type(s) of cell death in AD is not definitively known, and future studies will be required to definitively establish the cell death pathway(s) operating in our edited neurons.

### Differentiated Cultures Are Primarily Neuronal

Alzheimer’s disease has a well-known complexity with respect to the range of cell types associated with various disease-related observations (De Strooper and Karran, 2016). This cellular complexity can only be partially modeled in culture, primarily using long term organoid differentiation or co-culturing independently differentiated CNS cell types (Choi et al., 2014; Arber et al., 2017; Park et al., 2018). Our experimental objectives required not only long-term culture but also reliable and repeatable neuronal differentiation. Preliminary experiments using published protocols designed to generate AD relevant neurons proved too variable in our hands (Wicklund et al., 2010; Engel et al., 2016). We obtained much better consistency using a simple EB caudal hindbrain protocol originally developed to enrich for caudal motor neuron (Amoroso et al., 2013). Although many types of neurons are affected in AD, motor neurons are not. However, rostral differentiation of human AD mutant (APPV717I) iPSC cells using a similar differentiation protocol produced a population of neurons with clear amyloidogenic properties and only a modest decrease in amyloidogenic Aβ production relative to rostral differentiation of the same cells (Muratore et al., 2017).

RNAseq data confirmed the caudal nature of differentiated neurons based on expression of cell type-specific marker genes. A few rostral marker genes, however, were also expressed. Multiple neurotransmitter phenotype-specific genes were detected, but only low levels CHAT suggesting that cholinergic motor neurons are unlikely to be a prominent neurotransmitter phenotype as expected for motor neuron enrichment. Notably, only extremely low or undetectable levels of non-neuronal marker genes (primarily different types of glial cells) were expressed. This is consistent with weekly addition of mitotic blocker. We thus conclude that the phenotypes we observe are primarily neuronal and not likely related to other non-neuronal cell types such as microglia or astrocytes known participate in AD primarily through modulation of inflammatory pathways or removal of secreted Aβ. Neither of these processes appears relevant to our model. RNAseq data for cell-specific marker gene expression results are summarized in Supplementary Table S5.

### APOEe3/e4 Genotype

The neurons we studied all have an APOE e3/e4 genotype. The APOE e4 allele is the major genetic risk factor for non-familial AD estimated to be associated with 65–80% of all cases (Corder et al., 1993; Huang, 2010). Numerous studies have suggested both Aβ-dependent and independent cellular and molecular pathways associated with the e4 risk allele (Kim et al., 2009; Huang, 2010). Most are believed to operate through non-neuronal glial cell types (Wyss-Coray et al., 2003; Efthymiou and Goate, 2017) and modulate extracellular Aβ removal (Kim et al., 2009) and thus not likely to contribute to the phenotypes we studied. However, neuronal mechanisms are also known. For example, iPSC organoid cultures from a non-AD individual homozygous for e3 did not accumulate cellular Aβ after 6 months (Lin et al., 2018). When edited to homozygous e4, however, Aβ accumulation could be detected in an organoid extract. This study also established widespread neuronal-specific gene expression changes that were dependent on APOE allele type. An additional recent study demonstrated that homozygous e4 iPSC-derived neurons exhibit an Aβ-dependent toxic gain-of-function phenotype sufficient to result in ND of GABAergic neurons (Wang et al., 2018). Interestingly, this phenotype was species specific since it was not observed in similar mouse cultures. An APOEe4 fragment can also facilitate intracellular accumulation of Aβ42 (Dafnis et al., 2010) and APOE allele type has been associated with amyloid accumulation, especially during the early seeding stage (Liu et al., 2017). Taken together these observations suggest potential roles for e4 in intracellular neuronal Aβ retention when Aβ is produced by amyloidogenic processing of APP. Perhaps the primary significance of the e4 allele in our direct Aβ expressing cell lines is also to facilitate intracellular retention. This could also potentially explain the relatively high level of intracellular oligomer accumulation we observed relative to only modest intracellular accumulation in other AD culture models of unspecified APOE allele type (Kondo et al., 2013; Choi et al., 2014). Additional studies will be required to establish this directly.

### Synaptic Deficits

We documented a deficit in synapsin1-stained puncta in 34-day-old cultures which was specific for Aβ42-edited cells. This suggests a deficiency in the number of synapses relative to either Aβ40 or unedited cells at this culture age. Synaptic deficits are an early AD phenotype but have rarely been reported in human AD culture models (Mungenast et al., 2016). Other experimental models attribute synaptic deficits to increased amyloidogenic production of Aβ resulting in increased synaptic activity, or to a complex relationship to amyloidogenesis (sometimes involving non-Aβ40 or Aβ42 APP-derived proteolytic products) or even a tau dependence (Forner et al., 2017). The synaptic deficiency we observed is most likely a result of direct Aβ42 expression and subsequent accumulation of oligomeric/aggregated Aβ42. Unedited cells have only low-level accumulation of Aβ oligomer/aggregates and Aβ40-edited cells have significantly less than Aβ42 cells. Additionally, we do not observe significant tau-related phenotypes (see below). Importantly, we did not distinguish between a failure in synaptogenesis or a loss of existing synapses which would require additional experimental observations and we did not analyze synaptic activity.

### Phospho-Tau Phenotypes

While we did not systematically or extensively investigate Tau-related phenotypes in our model, we did observe that 62-day-old Aβ42-edited cultures had a similar staining intensity not significantly different from unedited cultures. We only used a single anti-phospho tau-specific antibody (targeting serine 244) (Grueninger et al., 2010). The distribution of phospho-tau staining did appear more somal in edited cells, but this was likely due to a prominent reduction in neurites (recognized by Tuj1 staining) at this late culture age. Increased tau phosphorylation is unlikely to be a prominent phenotype of direct Aβ42 expression. Additionally, RNAseq data for MAPT (the gene encoding tau) expression was similar for all three genotypes. These results are included in Supplementary Methods and Data and Supplementary Figure S6. One possible explanation for this absence of increased phospho-tau in edited cells is that the caudal neurons we studied are reportedly less susceptible to increased phospho-tau compared to more rostral neurons (Muratore et al., 2017). Further observations using additional anti-phospho tau antibodies and rostral differentiation protocols will be necessary to confirm this. A mechanistic role(s) for tau AD pathology as a driver of ND in AD is complex and still uncertain, beyond its end-stage pathologic importance (Ballatore et al., 2007). Interestingly, however, two recent stem cell studies, an APOEe4 model and a novel human-mouse hybrid model, appear to dissociate tau phenotypes from human-specific ND (Espuny-Camacho et al., 2017; Wang et al., 2018).

### Differential Gene Expression

The small number of DEGs we identified (93) generally show a more significant change with greater magnitude in Aβ42 compared to Aβ40 samples. This suggests that common pathways may be involved in these independently edited genotypes and agrees well with the exclusive and/or earlier or more penetrant phenotypic changes in Aβ42 samples at the time of mRNA isolation. It is challenging, however, to relate these expression changes to extensive whole transcriptome expression profiling of AD patient brain samples (or even other iPS cellular AD models). These studies often identify hundreds or thousands of DEGs (Castillo et al., 2017; Annese et al., 2018). This numerical difference with our results is likely due to multiple factors including the simplified rostral neuronal cell type we studied and the isogenic nature of our simplified culture model. Patient samples would contain significant genetic variance that may affect gene expression. They also contain variance in tissue and cell type sampling, co-morbidities, life style differences, penetrance of other AD phenotypes (i.e., tau pathology, non-neuronal inflammatory glial responses, etc.), all of which are likely to affect gene expression. Importantly, our cultures likely represent early changes in neuronal gene expression specific to direct Aβ-expression and accumulation of oligomeric intracellular Aβ.

Despite these comparative challenges, we note some selective and potentially interesting overlap with our data. For example, an AD vs. non-AD study of temporal cortex samples (Allen et al., 2016) revealed that 88% (82/93) are shared with our DEGs and 51% (42) have the same directional FC. Comparison with hippocampal study (Annese et al., 2018) identified 39% (36) genes with overlap and 67% (24) had the same directional negative FC (i.e., decreased expression).

Ingenuity Pathway analysis surprisingly identified “Increased Neuronal Loss” and “Decreased Memory” as the top scoring functional processes in our Aβ42-edited samples an obvious relevance to AD. Genes in these two functional processes overlap with each other. We illustrate this with a clustered sample level heat map in Supplementary Methods and Data and Supplementary Figure S7. IPA also suggested involvement of the “Neuroprotective THIOP1 Pathway in AD” as the only significant disease-related canonical pathway but only 2 of the 40 genes were differentially expressed. Since precise AD pathways related to Aβ-dependent ND are still largely unknown, this may be an expected result.

A surprising finding from our analysis was the identification of downregulated genes annotated related to cilia, an organelle not usually associated with AD. Five of these genes, however, were also downregulated in hippocampal AD patient samples (Annese et al., 2018) (DAW1, DNAH11, DNAI2, GDA, and TEKT1). Neurons usually contain a primary non-motile cilia (Guemez-Gamboa et al., 2014) believed to function as a major signaling center integrating environmental information through a wide variety of localized G-protein coupled and other types of neuroactive ligand receptors (Berbari et al., 2009). Interestingly, the KEGG “Neuroactive Ligand Receptor Interaction Pathway” (see Supplementary Table S4) was the top scoring pathway using GSEA analysis. Our results thus suggest that neuroactive signaling may be broadly disrupted in Aβ42 cultures and that this may be due to deficient ciliary function.

Ciliary functions are best studied in sensory neurons which contain specialized types of primary cilia. They have been implicated in olfactory neurons which exhibit Aβ-dependent connectivity defects (Cao et al., 2012), some types of retinal degeneration (Guemez-Gamboa et al., 2014) and hearing loss. In patients, hearing loss is also associated with AD and is more prominent in cognitively impaired older people (Uhlmann, 1989; Scheltens et al., 2016). Mutations in structural or functional ciliary genes are sufficient to cause a wide variety of distinctive developmental, cognitive, and degenerative phenotypes often involving the nervous system (Guemez-Gamboa et al., 2014). Neuronal cilia also play important roles in neurogenesis, axon guidance, establishment/maintenance of cell polarity, and even synaptic and memory functions (Lee and Gleeson, 2010; Berbari et al., 2014). Interestingly, cilia have a striking similarity to dendritic spines that include their protein and membrane composition as well as their receptive functions (Nechipurenko et al., 2013). Future experiments may be warranted to directly examine ciliary function in our culture model as well as in AD.

## Summary

Current AD therapeutic trials have failed at an unprecedented rate (Cummings et al., 2014, 2016; Schneider et al., 2014). These trials were primarily designed to disrupt APP amyloidogenic processing or remove extra cellular Aβ aggregates. Amyloidogenic pathway protease inhibitors, however, may also inhibit proteolysis of non-APP substrates necessary to support critical cellular functions. Our results suggest that removal of intracellular Aβ oligomers could be a more effective therapeutic target. This simple culture model could thus be an effective tool to identify such agents as well as discover molecular links between intracellular Aβ oligomer accumulation and the repertoire of downstream phenotypes we investigated.

## Data Availability Statement

The cell lines generated in this study will be made freely available to qualified investigators subject to completing a City of Hope Material Transfer Agreement. The sequencing data files have been deposited in the NIH GEO database (GSE119527).

## Author Contributions

PS: conceptualization, writing original draft, and funding acquisition. PS, TU, MM, SS, and CW: investigation. PS and JY: supervision. JY and SS: methodology. PS, CW, TU, MM, and JY: writing, reviewing, and editing. CW: formal analysis. TU and PS: visualization.

## Conflict of Interest

The authors declare that the research was conducted in the absence of any commercial or financial relationships that could be construed as a potential conflict of interest.

## Footnotes

1 https://www.alzforum.org/research-models/alzheimers-disease

2 http://changlab.uth.tmc.edu/gather/gather.py

3 http://software.broadinstitute.org/gsea/index.jsp

4 https://www.genecards.org
