# Supplementary material for: A Human Embryonic Stem Cell Model of Aβ-Dependent Chronic Progressive Neurodegeneration
Source: Front Neurosci. 2019 Sep 20;13:1007. doi: 10.3389/fnins.2019.01007 (PMC6763609; doi:10.3389/fnins.2019.01007)
Supplement: Supplementary file 1 [file Data_Sheet_1.docx]

Supplementary Methods and Data

# TALEN Editing

## Gene Editing (H9 Cells)


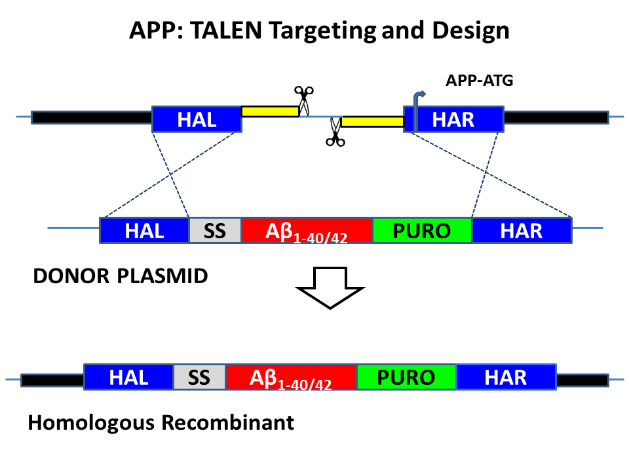


Figure 1. Schematic of TALEN editing strategy

The parental human ES cell line (WA09) was obtained from WiCell Foundation, Madison Wisconsin. All experiments were approved by the City of Hope Stem Cell Oversight Committee (#SC13002). Amyloid Precursor Protein (APP) gene was edited by using a TALEN (Transcription activator-like effector nuclease) pair and a donor template for homologous recombination. TALEN pairs were designed to cleave DNA just upstream of App translation start site as shown schematically in Fig. 1.

| **TALEN 1**  5’CGCCTGGCTCTGAGCCCC3’ |
| --- |
| **TALEN 2**  5’GCCGAGGAAACTGAC3’ |
| **TALEN 3**  5’CGGGCTCCGTCAGTTTCC3’ |
| **TALEN 4**  5’CCTCCGCGTGCTCTC3’ |

Figure 2. TALEN sequences

The TALEN design used criteria from Cermak et. al. (Cermak T, Doyle EL, Christian M, Wang L, Zhang Y, Schmidt C, et al. Efficient design and assembly of custom TALEN and other TAL effector-based constructs for DNA targeting. Nucleic Acids Res. 2011/04/16. 2011;39: e82. doi:gkr218 [pii] 10.1093/nar/gkr218). The TALEN pair sequences (Fig. 2) were assembled by using the Golden Gate assembly protocol from Addgene (Golden Gate TALEN and TAL Effector Kit 2.0 # 1000000024).

| **Rat preproenkephain secretory signal**: atggcgcagttcctgagactttgcatctggctcgtagcgcttgggtcctgcctcctggctacagtgcaggca |
| --- |
| **Aβ42**: gatgcagaattccgacatgactcaggatatgaa  gttcatcatcaaaaattggtgttctttgcagaagatgtgggttcaaacaaaggtgcaatcattggactcatggtgggcggtgttgtcatagcg |
| **Aβ40**: gatgcagaattccgacatgactcaggat  atgaagttcatcatcaaaaattggtgttctttgcagaagatgtgggttcaaacaaaggtgcaatcattggactcatggtgggcggtgttgtcatagcg |

Figure 3. Human Aβ and rat secretory signal sequences.

Donor templates were constructed with homology arms (HAL=563 bp; HAR=557 bp) from APP gene sequences ([ENST00000346798.7](http://uswest.ensembl.org/Homo_sapiens/Transcript/Exons?db=core;g=ENSG00000142192;r=21:27252861-27543446;t=ENST00000346798), Transcript ID) along with a secretory signal derived from the rat proenkephalin gene (PENK) and human Aβ40 or Aβ42 (Fig. 3).

To select for edited colonies, we used puromycin expression driven under PGK (Phosphoglycerate kinase) promoter. The sequence fragments for PGK-PURO were obtained from pMSCV puro plasmid (Clontech Cat. No. 634401).

| Primers for Genomic Insert Analysis | | |  |  |  |
| --- | --- | --- | --- | --- | --- |
| Target | Forward Primer | Sequence | Reverse Primer | Sequence | Amplicon |
| 5' Genomic Edit | 5' Recombinant F | GAAGTAAATGGGTTGGCCGCTTCTTTG | 5' Recombinant R | CCTGAGTCATGTCGGAATTCTGCATC | 712bp |
| 3' Genomic Edit | 3’ Recombinant F | GTCGAGGTGCCCGAAGGAC | 3’ Recombinant R | GGGCAACGATTCAAGAGCGA | 934bp |
| Left Homology Arm | LHA F | TTAACGCGGCCGCGGTTCGTTCTAAAGATAG | LHA R | AGCTGCAGAGATCTAGTCAGCTGATCCGGC | 563bp |
| Right Homology Arm | RHA F | AATATCTGCAGGGTACCAGCGGTAGGCGAG | RHA R | ATTTCTCGAGTGCTCCTTCCCCCTTCC | 557bp |

Figure 4. Primer sequences for screening correct genomic editing.

To generate edited H9 cells (Human embryonic stem cells: WiCell WA09), stem cells were cultured on feeder free system (Matrigel). The cells were harvested at appropriate confluency and Nucleofected with TALEN pair and donor template using an Amaxa Nucleofector. The nucleofected cells were allowed to grow on feeder free matrigel for 48 hours. The cells were then harvested and plated on puromycin resistant feeder cells at a dilution of 1/30. The cells were allowed to grow for 48 hours before changing to puromycin selection media. Selection was carried out for two weeks when appropriate size colonies were used for genomic PCR screening. The genomic screening was performed by harvesting ½ of a colony and extracting genomic DNA. This DNA was screened using specific primers spanning the homology arm and specific to the expected insert (Primers in Fig. 4) to confirm correct homologous recombination and insertion at the TALEN cutting site. The stem cell colonies with positive PCR amplicon at both ends were further characterized for edit specific Aβ expression using qRT-PCR analysis. Only colonies positive for genomic PCR analysis of both 3’ and 5’ junctions (Fig.5) and significant secretory Aβ expression were used for phenotypic analysis.


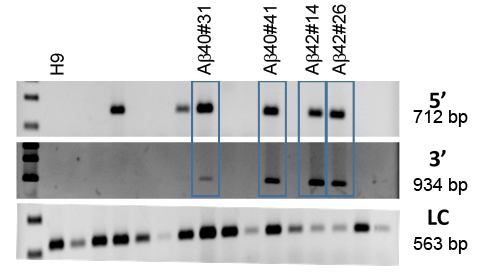


Figure 5. Genomic PCR to identify correct HR.

## Surveyor Assay

Surveyor Mutation detection kit from Transgenomic (Cat # 706025) was used to detect the efficiency of cleavage by the TALEN pairs. HEK293T cells were transiently transfected by Lipofectamine® 2000 (Invitrogen Cat# 11668-027). After 48 hours of transfection genomic DNA was extracted using QuickExtract^TM^ (DNA extraction solution was from epicentre an Illumina company (Cat# QE09050). The mutant and reference DNA were amplified using primers flanking the cleavage site (look primer file for sequences and conditions). Using thermal cycler Hetro and Homoduplexes were hybridized. Surveyor Nuclease was added to cleave the hybridization products and was used for fragment analysis on Agarose gel electrophoresis. Density of bands was used to determine the efficiency of TALEN cleavage (Fig. 6).

## Stem Cell Nucleofection


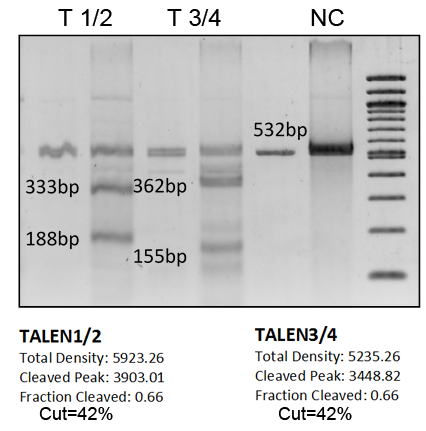


Figure 6. Surveyor Assay for TALEN pairs.

To increase survival stem cells were treated with 10 µM rock inhibitor (Stemgent Y-27632) at least 1 hour before starting nucleofection. Four wells of confluent H9 stem cells were grown on feeder free matrigel system in media (Stem Cell Technologies: mTeSR1 Cat# 05850) for 4 days. Media was changed daily and differentiating colonies were manually removed. All DNA samples (TALEN pairs and donor template) were purified using endotoxin free kits. Total DNA for TALEN pairs and donor template was ~5 μg per reaction in a total volume of 10 μl. For each sample we added 82 μl of Nucleofector® Solution 2 plus 18 μl of supplement 1 to make 100 μl of total reaction volume. Nucleofection solution was mixed with TALENs and Donor Template room temperature.

We used the Amaxa nucleofector following their recommended reagents and protocols. Briefly, H9 cells colonies were dissociated into single cells using accutase (7 min at 37^o^C), resuspended in ES cell media (DMEM F-12, 20% KO serum, Pen/Strep, NEAA, Glutamax, FGF, β-ME) and pelleted by centrifugation (1000 rpm for 5 min). Cells were resuspended in 1-2 ml of mTeSR medium with rock inhibitor, an aliquot was taken for counting and 1.5x10^6^ cells were used for each nucleofector sample. Cells were pelleted (850 rpm, 115 g, 3 min.), media was removed and the pellet resuspended in 100 μl of RT Nucleofector solution 2 along with the Talen/Donor template mix in a nucleofector cuvette and processed using program B-016 (Human Stem Cells). The sample was recovered in 0.5 ml of pre-warmed mTeSR medium (containing 10 μM Rock inhibitor) and were transferred to a matrigel (BD bioscience Cat #354230) coated 24-well plate. Cells were grown for two days, resuspended and plated on puromycin resistant feeder cells. Puromycin drug selection was initiated after 2 days and maintained for 14 days. All non-nucleofected cells died after two weeks under drug selection.

## qRT-PCR analysis

Total RNA was isolated from cells using RNA STAT60 RNA extraction reagent from Amsbio or Zymo Research. Where necessary RNA was concentrated using glycogen precipitation. For cDNA synthesis RETROscript® Reverse Transcription Kit (AM1710) or Quanta (Qiagen) was used. The cDNA was amplified using oligo (dT) primers. The cDNA generated was diluted and used for qRT-PCR analysis with iQ™ SYBR Green Supermix from BIORAD (1708880) in CFX96 machine. Mean normalized expression values were calculated relative to GAPDH reference gene. Cycling Conditions were: Denaturation/enzyme activation 95°C for 3 minutes; Denature 95°C, 30 seconds; annealing and extension 55°C for 20 seconds, 40 cycles. Melting curve: 55°C to 95°C for 5 seconds in 0.5°C increments to ensure a single amplicon.

# Microscopy and Image Analysis

## Microscopy

Fluorescent immunocytochemically stained cells were observed with a Zeiss Axio Observer microscope (Xenon illumination) using either a 20X NA=0.80 plan-apochromat objective or a 40x or 63x plan-apochromat objective (NA=1.4, Oil). Optical Z section images were acquired with a Zeiss Axiocam506 camera (0.05-1 μm spacing, total focal range spanning the thickness of the cultured cells, usually ~10-20 μm) using Zeiss Zen Blue microscope control software (SP2). Unstained cultures were observed using a Nikon Diaphot inverted microscope equipped with Hoffman modulation contrast objectives (HMC EF 10X NA=0.25 or HMC 20X LWD NA=0.4) and images were obtained with a SPOT RT230 cooled CCD camera operated by SPOT Advanced Imaging Software. For fluorescence live/dead analysis and accompanying modulation contrast images, Z sections were acquired at ~1 μm spacing spanning the whole culture depth. All image analysis was performed using semi or fully automated macros implemented in the FIJI version of NIH ImageJ (v1.46 or 2) ^30^. For visual clarity some images are adjusted for brightness and contrast using Adobe Photoshop (CS4 or CS5).

## Image Analysis

Image analysis was performed using the FIJI version of NIH ImageJ (v1.46 or 2) (https://imagej.net/Fiji). Control images were collected for samples with no addition of either primary antibody or secondary antibody.

**DAPI**: Due to variability in the size of neuronal clusters both within a particular differentiation and across the different genotypes as well as across independent differentiations, quantitative data were normalized to the number of DAPI stained nuclei. Briefly, the DAPI channel was processed using the rolling ball (radius =50) background subtract function, autothresholded (Otsu method), converted to an inverted binary image and individual nuclei were estimated following a dilation and watershed operation. Counts were obtained with the analyze particles function using a filter size >30 µm2 and a circularity between 0 and 0.85, excluding nuclei on the edges of the image. In some cases, 2-3 optical sections were combined using a maximum intensity Z projection of individual adjacent optical sections.

**DCX and NeuN**: Because of their complex shapes, doublecortin stained neurons were counted manually using the green cannel from the corresponding optical section or Z projection. NeuN quantification used the red cannel background subtracted images (rolling ball radius = 400, size = 60µm, circularity 0-0.9). Multiple neuronal clusters were analyzed for three independent differentiations for each genotype and normalized to the number of DAPI stained nuclei.

**Aggregated Aβ**: Oligomeric/aggregated Aβ (7A1a) quantification was done by creating a tightly bound box around each individual neuronal cluster and cropping the image to this area. Background was subtracted using a 150-pixel rolling ball radius. A maximum intensity projection was then created from an image stack (0.5μm Z intervals, 16-60 images), then using the Z-projection maximum intensity algorithm. The individual color channels were separated and the Aβ channel was thresholded using the IsoData method to measure the area of positive staining within the neural cluster and expressed as a percentage of the total cluster area. Neuronal cluster area was measured from Hoffman interference contrast images by using the freehand tool to manually trace the edges of clusters and using the measure area function.

**Synapsin 1**: Synapsin1 puncta quantification was done by subtracting the background using a 150-pixel rolling ball radius and creating a maximum intensity projection of a stack of images (0.5µm sections, 11-31 images). The nuclei were counted the same way as in the DCX and NeuN analysis and the synpasin1 puncta were counted using the ImageJ find maxima function. The number of synapsin1 puncta were then normalized to the number of DAPI nuclei present in each NC.

**Lamp1, Rab5, Rab3A, LC3B**: Coverslips from 38-43 or 63-day old cultures were stained with anti-Lamp 1, anti-Rab5, anti-Rab3A or anti-LC3B antibody. Particle counts were measured following background subtraction (rolling ball radius = 15 pixels) and auto thresholding using a maximum intensity Z projection of two or three 1 µm spaced optical sections with the analyze particles function (size filter = 0.05-2 µm^2^) and normalized to the number of DAPI stained nuclei.

**Live-Dead Assay**: Live/dead analysis was done by staining neuronal clusters growing on coverslips or in 12 well plates using ethidium homodimer (red fluorescence, dead cells) and calcein AM esterase substrate (green fluorescence when hydrolyzed) (ThermoFisher, #R37601). Three to five individual focal planes were obtained along with a corresponding Hoffman contrast image. The number of fluorescent pixels in an area was measured by separating individual color channels of the stack, subtracting background using a 50-pixel rolling ball radius and creating a maximum intensity projection using auto brightness and contrast to threshold each channel. Data is presented as percent red area relative to the total area of red plus green channels. Hoffman modulated contrast images were obtained as a Z-stack with a spacing of ~1 μm and processed using the simple EDF wavelet processing Plugin in FIJI (Easy setting, medium quality) to construct an extended depth of field image.

# RNA-Seq

Reads were aligned against the human genome (hg19) using TopHat2 [1]. Read counts were tabulated using htseq-count [2], with UCSC known gene annotations (TxDb.Hsapiens.UCSC. hg19.knownGene, [3]). Fold-change values were calculated from Fragments Per Kilobase per Million reads (FPKM, [4]) normalized expression values, which were also used for visualization (following a log2 transformation). Aligned reads were counted using GenomicRanges [5]. P-values were calculating from raw counts using edgeR [6], and false discovery rate (FDR) values were calculated using the method of Benjamini and Hochberg [7]. Prior to p-value calculation, genes were filtered to only include transcripts with an FPKM expression level of 0.1 (after a rounded log2-transformation) in at least 50% of samples [8] as well as genes that are greater than 150 bp.

Two differential steps were used to define differentially expressed genes. First, genes that vary between Aβ42 and parental H9 cells were identified using a 2-variable differential expression model (Aβ42 status, and run/batch), with the initial set of genes were identified as differentially expressed if they had a |fold-change| > 1.5 and FDR < 0.25. Then, a set of differentially expressed genes between Aβ40 versus parental H9 were identified as differentially expressed if they could be identified with a more liberal criteria (|fold-change| > 1.2, and unadjusted p-value < 0.05); if there was overlap between genes with the same fold-change sign, those overlapping genes were filtered. The remaining genes were then used for visualization in a batch/run-centered heatmap (expression centered by batch, prior to setting the per-gene expression to have a mean to 0 and standard deviation to 1), using heatmap.3 (https://github.com/obigriffith/biostar-tutorials/blob/master/Heatmaps/heatmap.3.R) and Pearson’s Dissimilarity as the distance metric.

Gene Ontology (GO [9]) enrichment was calculated using goseq [10].

Systems-level analysis was performed in IPA (Ingenuity® Systems, www.ingenuity.com, accessed on 5/26/2017) and GATHER ([11]) accessed on 5/26/2017).

# Cell culture and differentiation

Stem cell differentiation was adapted from: Amoroso MW, Croft GF, Williams DJ, et al. (2013) Accelerated high-yield generation of limb-innervating motor neurons from human stem cells. *J Neurosci*. **33,** 574-586. doi:10.1523/JNEUROSCI.0906-12.2013.

## ES cell culture and maintenance

ES cell lines (H9 or edited H9) are cultured on gelatin coated (0.1% in PBS) wells with a feeder layer of irradiated mouse embryonic fibroblasts (Fisher Scientific, A34180). Stem cells are cultured in HuES medium (after the second day of plating, media is changed daily). Cells were observed daily and any colonies that start to differentiate were removed using a sterile pipette. ES colonies should have perfect undifferentiated colony morphology before beginning differentiation. Differentiation was started ~1 week after the previous passage (i.e. cells maintained in culture were passaged ~every week). Approximately 8-9 confluent wells of a 6-well dish of stem cells are sufficient to make one 10 cm dish of EB bodies.

## EB Generation

Day 0: Colonies were released from fibroblast attachment using dispase (1 mg/ml) in PBS. Aspirate old medium and add dispase solution. Incubate in tissue culture incubator until colonies begin to detach with gentle tapping (colonies should begin to curl up from the edges and detach from CF-1 MEFs (usually after ~12-15 minutes, observed at regular intervals with microscope). When colonies are detaching add HuES media to stop the dispase digestion. Remove dissociated colonies by gentile pipetting with a 10 ml pipette and transfer to a 50 ml falcon tube. Let cells settle for ~3-5 min and wash gently with ~10 ml PBS (2x). If all the colonies were not released from the plate repeat above procedure gently using a cell scraper to increase the yield. In general, ~95% of colonies were recovered. Triturate released colonies in 1 ml of HuES supplemented with additional factors. Use a P1000 pipette and triturate ~7-10 times. Resuspend the dissociated cells in HuES medium supplemented with additional factors (20 ng/ml FGF-2, 10 μM SB431542, 0.2 μM LDN193189 (Stemgent) and 20 μM Y-27632 (ROCK inhibitor, Stemgent). Pipette cell suspension into a 10 cm culture plate containing 9 ml of HuES with additional factors. The plating concentration should be ~400,000 cells/ml. This is considered day 0 for EB production.

## Preparation of EBs

1. Day 2: Change media. Collect cells in a 15 ml tube, centrifuge for 2 min at 100xG (1000 rpm). Aspirate media and replace with fresh HuES + factors medium (Do not pipette up and down, you want to keep small intact EB).
2. Day 3: collect as above and change to Neural Induction Media 1(NIM 1).
3. Day 5: change media to NIM 2. (NIM1+ Ascorbic Acid (AA, Sigma) 0.4 μg/μl, + all trans-retinoic acid (RA, Sigma) 1 μM and – Y-27632 ROCK Inhibitor).
4. Day 7: change media to NIM3 (NIM1+SAG, –βFGF).
5. Day 9-15: NIM + BDNF + AA +RA + SAG as above, Change every 3 days.
6. Day 17 to 28-31 days (most often 28d): Switch to NDM 1 medium changed every 3 days. Maintain all supplements as above plus: B-27 (50x, Invitrogen), GDNF (10 ng/mL, R&D systems), CNTF (10 ng/mL, R&D systems), IGF-1 (10 ng/mL, R&D systems).

## Neuronal differentiation

1. Dissociate differentiated EBs at day 20-31 (Generally at 28 days).
2. Collect EBs in 50ml Falcon tube and allow to settle for 5 minutes.
3. Rinse EBs and let settle in CMF (Calcium and Magnesium free PBS).
4. Dissociate into individual cells using 2 ml of 0.25%Trypsin-EDTA (GIBCO 25200-056). Incubate 37^°^ ~6-7 min while gently swirling in 37^o^ water bath (critical step: do not over incubate).
5. Add an equal volume FBS to stop the trypsin digestion and then 8 ml of CTWM.
6. Centrifuge 3 min at 1400 rpm (IEC Centra CL2 centrifuge)
7. Remove supernatant and resuspend pellet in 1ml CTWM by trituration ~7 passes with p1000 pipette (often requires vigorous up and down pipetting) and add 10 ml CTWM.
8. Pass cells through a 40 µm strainer to remove large clumps (Sigma-Corning CLS431750-50EA) into a new 50 ml tube.
9. Take a sample for cell counting.
10. Centrifuge at 1600 rpm.
11. Resuspend single cell suspension in NDM medium + all supplements+ β-ME (25 μM) + Glutamate (25 μM)
12. Seed dissociated cells at 1.7x10^6 per well in a 6 well plate or 0.34x10^6 per well in a 24 well plate coated with poly-L-ornithine and laminin. Culture with EdU to maintain only post-mitotic cells.
13. Unused cells can be cryopreserved by freezing in 2x freezing medium (Chemicon, for ES cells) with Frosty chambers (Fisher) optional.

## Media Recipes

General Notes: BDNF, GDNF, IGF-1, CNTF lyophilized powders are dissolved in 0.1% BSA that has been filtered through a 0.22um filter. BDNF 10ug powder + 200ul BSA = 50µg/ml stock, GDNF 10ug powder + 200ul BSA = 50µg/ml stock. IGF-1 100ug powder + 2ml BSA = 50µg/ml stock. CNTF 20ug powder + 1ml BSA = 20µg/ml stock. Ascorbic Acid powder is mixed with filtered, autoclaved ultrapure water. Retinoic acid powder is dissolved in DMSO – protect from light. To make 1mM stock: add 10µl of 100mM to 990 µl of DMSO. To make 100 mM from powder: add 1.67ml DMSO to 50mg.

### HuES Medium

| Final Concentration | Stock | Volume for 10ml solution |
| --- | --- | --- |
| 80% DMEM-F12 | 1x | 7.67ml |
| 20% KO Serum Replacer | 1x | 2ml |
| 50 U and 50 mg/ml Pen/Strep solution | 100x | 100μl |
| 1% Non-essential Amino Acids | 100x | 100μl |
| 1 mM L-Glutamax | 100x | 100μl |
| 0.1 mM β-mercaptoethanol | 100mM | 10µl |
| 20 ng/ml β-FGF | 10μg/ml | 20μl |

Notes: β-ME and βFGF must be added fresh on the day of use. Other components can be mixed together and stored in refrigerator until use. β-mercaptoethanol: 7 ul of 14.3 M β-ME + 1 ml calcium and magnesium free PBS. (Protect from light and make a new stock every week stored at -20. βFGF: 50 μg dissolved in 5 ml Knock-Out Serum (Life Technologies 10828-028) to make a 10 μg/ml stock.

### EB1 (HuES +βME, βFGF, ROCK inhibitor, SB431542)

| Final Concentration | Stock | Volume for 10ml solution |
| --- | --- | --- |
| 80% DMEM-F12 | 1x | 7.65ml |
| 20% KO Serum Replacer | 1x | 2ml |
| 50 U and 50 mg/ml pen/strep solution | 100x | 100μl |
| 1% Non-essential Amino Acids | 100x | 100μl |
| 1mM L-Glutamax stock | 100x | 100μl |
| 0.1 mM β-mercaptoethanol | 100mM | 10μl |
| 20 ng/ml βFGF | 10μg/ml | 20μl |
| 10μM Y-27632 (ROCK inhibitor) | 10mM | 10μl |
| 10μM SB431542 | 10mM | 10μl |
| 0.2μM LDN193189 | 10mM | 0.2μl |

Notes: ROCK inhibitor, SB431542, and LDN193189 stocks come in solution. LDN is viscous, be careful. βFGF helps with single cell survival, SB and LDN are “stemness” inhibitors.

### NIM1 (Neural Induction Medium, EB1 media-KO serum, -βME, + heparin)

| Final Concentration | Stock | Volume for 10 ml solution |
| --- | --- | --- |
| 99% DMEM-F12 | 1x | 9.55ml |
| 50 U and 50 mg ml-1 pen/strep solution | 100x | 100μl |
| 1% Non-essential Amino Acids | 100x | 100μl |
| 1mM L-Glutamax | 100x | 100μl |
| 1 x N2 Supplement | 100x | 100μl |
| 2 μg/ml Heparin | 2mg/ml | 10μl |
| 20 ng/ml βFGF | 10μg/ml | 20μl |
| 10 μM Y-27632 (ROCK inhibitor) | 10mM | 10μl |
| 10 μM SB431542 | 10mM | 10μl |
| 0.2 μM LDN193189 | 10mM | 0.2μl |

### NIM 2 (NIM1 -ROCK inhibitor, +AA, +RA)

| Final Concentration | Stock | Volume for 10ml solution |
| --- | --- | --- |
| DMEM-F12 | 1x | 9.56ml |
| 50 U and 50 mg ml-1 Pen/Strep solution | 100x | 100μl |
| 1% Non-essential Amino Acids | 100x | 100μl |
| 1mM L-Glutamax | 100x | 100μl |
| 1 x N2 Supplement | 100x | 100μl |
| 2 mg/ml Heparin | 2mg/ml | 10μl |
| 10 ng/ml BDNF | 50μg/ml | 2μl |
| βFGF 20 ng/ml | 10 μg/ml | 20μl |
| 0.4 mg /ml Ascorbic acid | 10mg/ml | 0.4μl |
| 1 µM RA | 1mM | 10μl |

Notes: RA should be used from stock within 6 months of storage in -80; aliquot and store in light protected environment, store as single use aliquots.

### NIM 3 (NIM2 + SAG, -FGF)

| Final Concentration | Stock | Volume for 10ml solution |
| --- | --- | --- |
| DMEM-F12 | 1x | 9.56 ml |
| 50 U and 50 mg ml-1 pen/strep solution | 100x | 100μl |
| 1% Non-essential Amino Acids | 100x | 100μl |
| 1mM L-Glutamax | 100x | 100μl |
| 1 x N2 Supplement | 100x | 100μl |
| 2 mg/ml Heparin | 2mg/ml | 10μl |
| 10 ng/ml BDNF | 50μg/ml | 2μl |
| 0.4 mg /ml Ascorbic acid | 10mg/ml | 0.4μl |
| 1 µM RA | 1mM | 10μl |
| 2 mM SAG | 10mM | 2μl |

### NDM 1 (Neural Differentiation Medium)

| Final Concentration | Stock | Volume for 10ml solution |
| --- | --- | --- |
| Neural Basal Medium | 1x | To 10ml |
| 50 U and 50 mg ml^-1^ pen/strep solution | 100x | 100μl |
| 1% Non-essential Amino Acids | 100x | 100μl |
| 1mM L-Glutamax | 100x | 100μl |
| 1 x N2 Supplement | 100x | 100μl |
| 10 ng/ml BDNF | 50μg/ml | 2μl |
| 0.4 μg/ml Ascorbic acid | 10mg/ml | 0.4μl |
| 1 μM RA | 1mM | 10μl |
| 2 μM SAG | 10 mM | 2μl |
| 1x B27 supplement | 50x | 200 μl |
| 10 ng/ml GDNF | 50μg/ml | 2 μl |
| 10 ng/ml CNTF | 20μg/ml | 5 μl |
| 10 ng/ml IGF-1 | 50μg/ml | 2 μl |

Notes: Before dissociating cells coat coverslips with poly-L-ornithine and laminin (requires 2 days). Poly-L-ornithine – (coated first) stock is 50 mg/ml: dilute with COLD DMEM/F12, working concentration is 0.1 mg/ml (2.0 μl for 1 ml). Incubate overnight at 37C. To make stock from powder: dissolve 100mg in 2ml of autoclaved DDW and pass through 0.22 μm filter = 50mg/ml. Laminin – (coated second) stock is 1 mg/ml: dilute with COLD DMEM/F12, working concentration is 20 μg/ml (20ul per 1 ml). Incubate overnight at 37C.

### CTWM (Complete Trituration Wash Media, need ~20mL per genotype)

| *Final Concentration* | *Stock* | *Volume for 100ml solution* |
| --- | --- | --- |
| 1x PBS (calcium & magnesium Free) | 1x | 89.1ml |
| 25mM Glucose | 1M | 2.5ml |
| 0.1% dialyzed BSA | 4% | 2.5ml |
| N2 supplement | 100x | 1ml |
| B27 supplement | 50x | 2ml |
| MgCl2 2mM | 1M | 200μl |
| EDTA 1mM | 0.5M | 200μl |
| FBS | 1x | 2.5ml |

# Maintenance of Differentiated Neurons

## NDM (Neural Differentiation Medium)

| Final Concentration | Stock | Volume for 10ml solution |
| --- | --- | --- |
| Neural Basal Medium | 1x | 9.38 ml |
| 50 U and 50 mg ml^-1^ pen/strep solution | 100x | 100μl |
| 1% Non-essential Amino Acids | 100x | 100μl |
| 1mM L-Glutamax | 100x | 100μl |
| 1x N2 Supplement | 100x | 100μl |
| B27 supplement | 50x | 200μl |
| 10ng/ml BDNF | 50μg/ml | 2μl |
| 10ng/ml GDNF | 50μg/ml | 2μl |
| 10ng/ml IGF-1 | 50μg/ml | 2μl |
| 10ng/ml CNTF | 20μg/ml | 5μl |
| 0.4μg/ml Ascorbic acid | 10mg/ml | 0.4μl |
| 25μM Glutamate | 25 mM | 1μl |
| 25μM β-mercaptoethanol | 100mM | 2.5μl |
| 1µM RA | 1mM | 10μl |
| 0.μm EdU | 5mM | 1ul |

Notes: Glutamate: Sigma G8415-100G, M.W. 147.13, prepare 25 mM stock (0.11 g and dissolve in 30 ml of 1N HCL). EdU (mitotic poison) is used to keep dividing cells from over-growing the culture. A 5 mM stock solution is diluted in NDM to a final concentration of 0.5 μm. The initial EdU treatment is for exactly 24 hrs before changing back to NDM with no EdU. After this initial treatment, cultures are treated with EdU for 24 hrs every week until cultures are approximately 50 days post-EB dissociation.

# Antibodies

| Antibody Name | Type | Supplier | Cat. Number | Concentration |
| --- | --- | --- | --- | --- |
| Primary antibodies: |  |  |  |  |
| Anti-Doublecortin | Rabbit polyclonal | Abcam | ab104224 | 1μg/ml |
| Anti-NeuN | Mouse monoclonal | Abcam | ab104224 | 2μg/ml |
| 7A1a (Anti-Aβ oligomeric/aggregate specific) | Mouse monoclonal | New England Rare Reagents | ALI 2b13 | 1.4μg/ml |
| Anti-Aβ 6E10 | Mouse monoclonal | Covance | SIG-39320 | 1mg/ml |
| Anti-Tuj1 (anti-beta III tubulin) | Mouse monoclonal | Abcam | ab78078 | 1μg/ml |
| Anti-Synapsin 1 | Rabbit polyclonal | Abcam | ab8 | 0.2μg/ml |
| Anti-LC3B | Rabbit polyclonal | Cell Signaling |  | 1:400 |
| Anti-Rab3A | Rabbit polyclonal | Abcam | ab3335 | 1:200 |
| Anti-Rab5 | Rabbit polyclonal | Abcam | ab18211 | 1μg/ml |
| Anti-Lamp 1 | Rabbit polyclonal | Abcam | ab24170 | 0.25μg/ml |
| Anti-Nestin | Rabbit polyclonal | Abcam | ab92391 | 1:250 |
| Anti-Phospho-tau(S422) | Rabbit polyclonal | Abcam | ab79415 | 1:200 |
| Anti-Oct4 | Goat polyclonal | Abcam | ab27985 | 2μg/ml |
| Anti-GFAP | Chicken polyclonal | Abcam | ab48050 | 1:500 |
| Anti-Choline acetyltransferase | Rabbit polyclonal | Abcam | ab68779 | 1:500 |
| Anti-Rab4 | Rabbit monoclonal | Abcam | ab109009 | 1:500 |
| Secondary antibodies: |  |  |  |  |
| anti-Chicken IgY H&LAlexa Flour 488 | Goat polyclonal | Abcam | ab150169 | 3.9μg/ml |
| anti-Rabbit IgG H&L Alexa Flour 488 | Goat polyclonal | Abcam | ab150077 | 2μg/ml |
| anti-Mouse IgG H&L Alexa Flour 594 | Goat polyclonal | Invitrogen | a11005 | 2μg/ml |

# Primers

## qRT-PCR

| Target | Forward (5’→3’) | Reverse (5’→3’) |
| --- | --- | --- |
| GAPDH | ATGGGGAAGGTGAAGGTCGGAGTC | GGGATTTCCATTGATGACAAGCTTCCCG |
| Edited Rat SS-Aβ* | ATGGCGCAGTTCCTGAGA | ATGATTGCACCTTTGTTTGAACC |
| Edited Rat SS-Aβ42** | ATGGCGCAGTTCCTGAGA | CGCTATGACAACACCGC |
| APP Exon 1/2 | GTTTGGCACTGCTCCTGCTG | CTGATGGATCTGAATCCCACTTCCC |
| APP Exon 5/6 | GAGGAGGATGACTCGGATGTCTGG | GGTGGTTCTCTCTGTGGCTTCTTCGT |
| APP Exon 16/17 | GATGCAGAATTCCGACA | CCATGATGAATGGATGTGTAC |
| RAB5 | CTAGTGCTTCGTTTTGTGAAAGG | CATATACAACTATGGCTGCTTGTG |
| LAMP1 | CTACTGCTGTTGCTGCTGCTCG | GGCATCTGATGGCAGGTCAAAGG |
| LC3B | ATGCCGTCGGAGAAGACCTTCAAGCAG | CTTGATGAGCTCACTCATGTTGACATGGTCAGG |
| VAMP7 | CTGGAGGTGACAGAGCAGATT | TGTCTGTGCTCTTGAACCGT |
| RAB11 | GACGACGAGTACGACTACCTC | GCAAACTCTACTCCAATGGTGC |
| PICALM | AGCAAGTACATGGGGAGATGC | TTAAGGCCAGCTGAAGGGTG |
| RAB3A | AAAGTCACCGCCGCTAGG | ACCTTGAAGTCGATGCCCAC |
| *Rat secretory signal sequence (Forward) and human Aβ40 or 42 (Reverse)  **Ab42 edit specific reverse primer | | |

## Genomic

| Target | Forward (5’→3’) | Reverse (5’→3’) |
| --- | --- | --- |
| 5' Recombinant insert | GAAGTAAATGGGTTGGCCGCTTCTTTG | CCTGAGTCATGTCGGAATTCTGCATC |
| 3’ Recombinant insert | GTCGAGGTGCCCGAAGGAC | GGGCAACGATTCAAGAGCGA |
| Left homology arm | TTAACGCGGCCGCGGTTCGTTCTAA AGA TAG | AGCTGCAGAGATCTAGTCAGCTGATCCGGC |
| Right homology arm | AATATCTGCAGGGTACCAGCGGTAGGCGAG | ATTTCTCGAGTGCTCCTTCCCCCTTCC |

# Supplemental Figures S1-S6


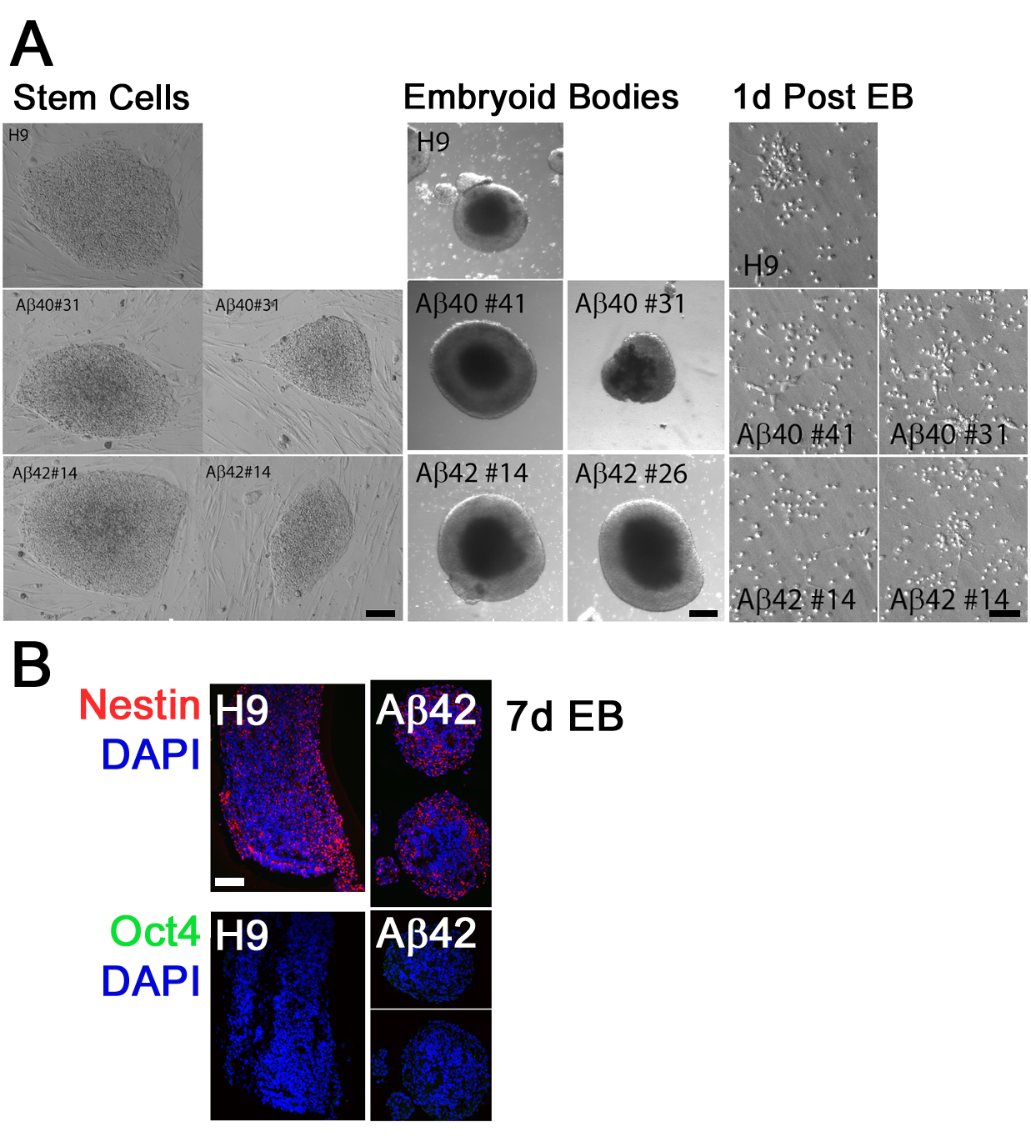


**Fig S1**. A. Representative morphological appearance of stem cell cultures, embryoid bodies (28-day old) and 1-day old cells in neuronal differentiation media. Scale bar = 500 μm, stem cells; 50 μm, EBs, 10 μm, neurons. B. Immunocytochemical staining of sectioned 7-day old embryoid bodies. Red=anti-Nestin, green=anti-Oct4, Blue=DAPI.


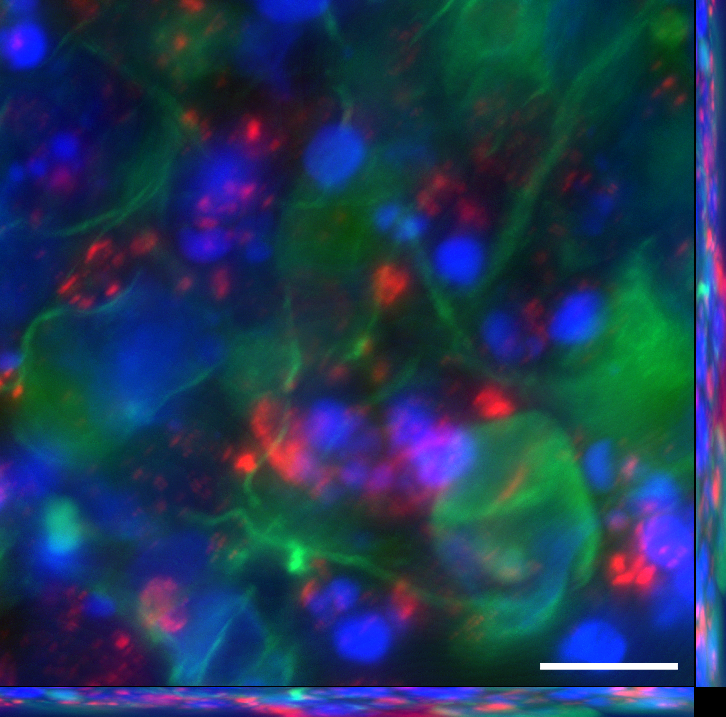


**Fig S2. Orthogonal projections of 7A1a staining indicate a primarily intracellular accumulation.** A maximum intensity projection of a 32-day old neuronal cluster from an Aβ42 edited culture (main image). XZ (bottom) and YZ (right) projection images of the same image stack are shown on the bottom and right. The red 7A1a positive staining (aggregated/oligomeric Aβ) is primarily near blue (DAPI) stained nuclei or pyknotic nuclei and largely within the limits of green (Tuj1) positive neuronal staining. Scale bar is 10 μm. **Method**: A 60 optical slice stack (0.07 μm spacing) was initially processed in FIJI to correct for uneven illumination using the CIDRE plugin [Smith, K., Li, Y., Piccinini, F., Csucs, G., Balazs, C., Bevilacqua, A., & Horvath, P. (2015). CIDRE : an illumination- correction method for optical microscopy. Nature Methods, 12(5), 404–406. <https://doi.org/10.1038/NMETH.3323>]. The image was cropped and a maximum intensity projections and orthogonal projections generated in FIJI using a 30 slices sub stack (2.1 μm of total depth). The final image was assembled in Adobe Photoshop (CS4) and adjusted for brightness and contrast.


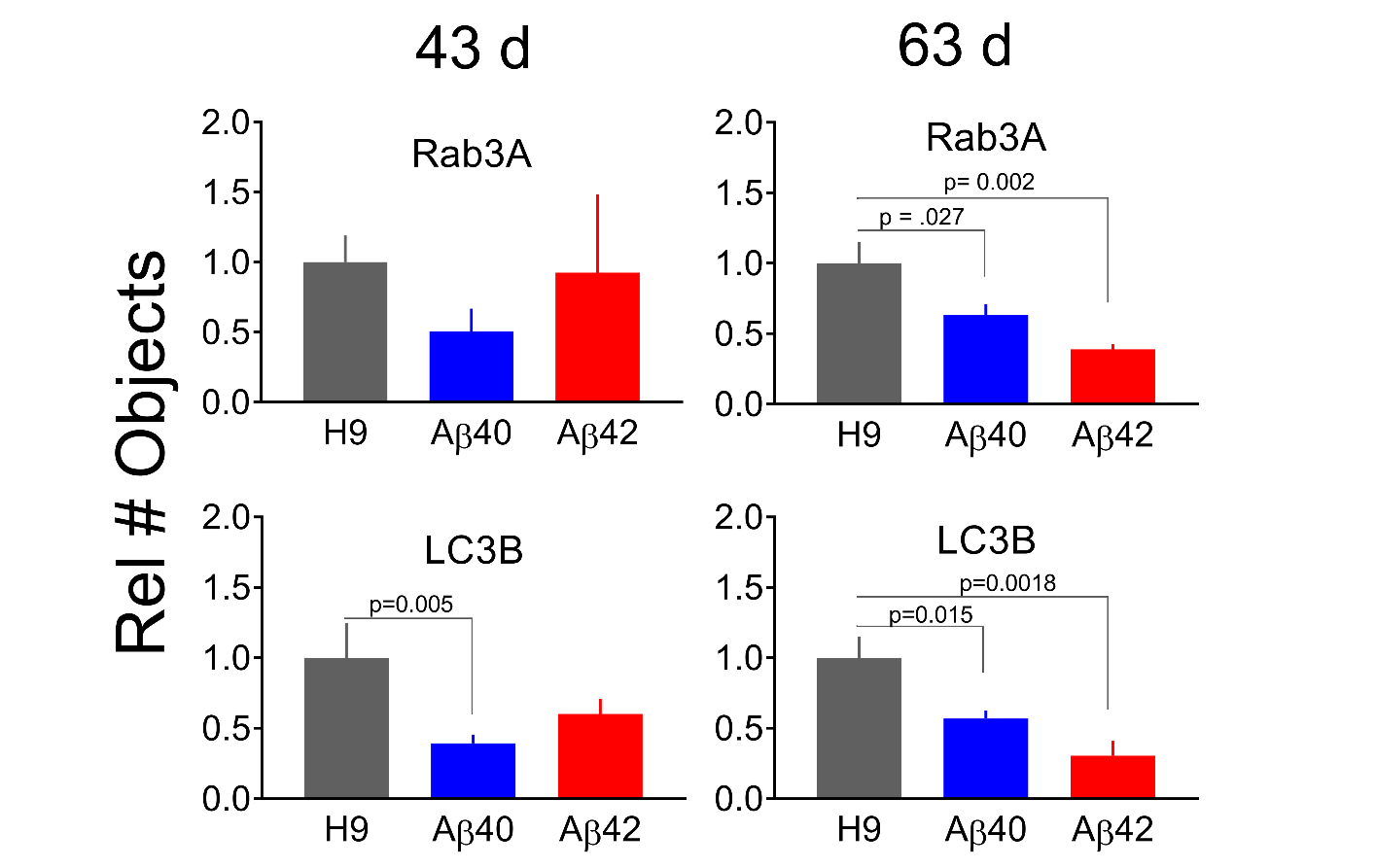


**Fig S3.** The relative number of Rab3A and LC3B puncta were more variable in individual samples of all genotypes and both decreased primarily in older cultures. Individual NCs from 2 independent differentiations were stained with either anti-Rab3A (synaptic vesicle associated marker) or LC3B (autophagosome marker) antibody. There was a significant decrease in LC3B objects in Aβ40 samples at 43 days and a decrease in both Aβ40 and Aβ42, as well as LC3B puncta, in 63-day cultures (ANOVA, Dunnett corrected). Bars are mean ±SEM, N=5-20). Scale bar = 20 μm.


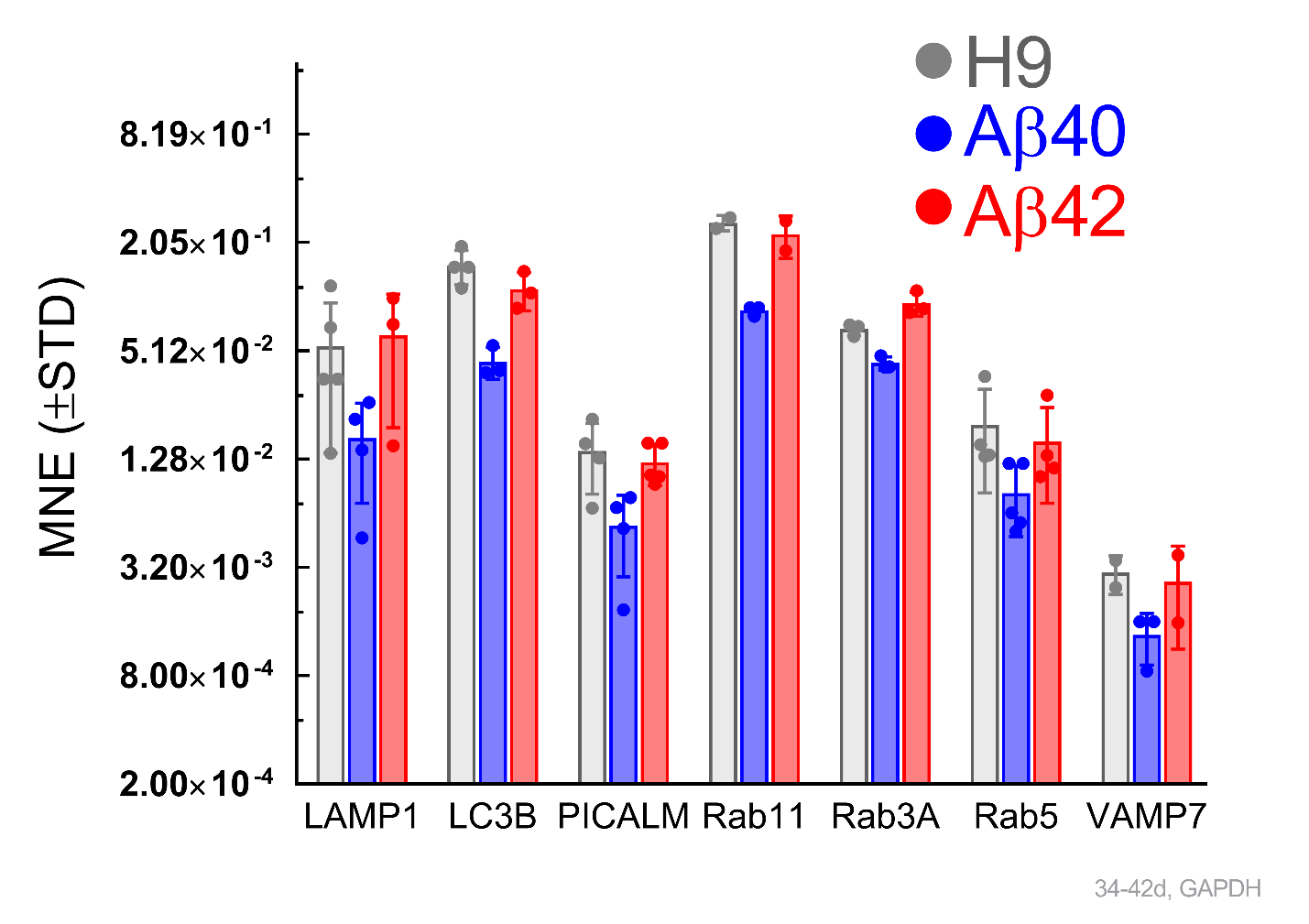


**Fig S4**. **Expression levels of selected vesicular genes**. qRT-PCR analysis (Syber green) of selected vesical associated marker genes. Mean normalized expression values relative to GAPDH. Individual points are samples taken from independent differentiations. There were no significant differences in the level of expression for these genes among the 3 genotypes (ANOVA, Dunnett corrected). Cultures were 34-42 days old.


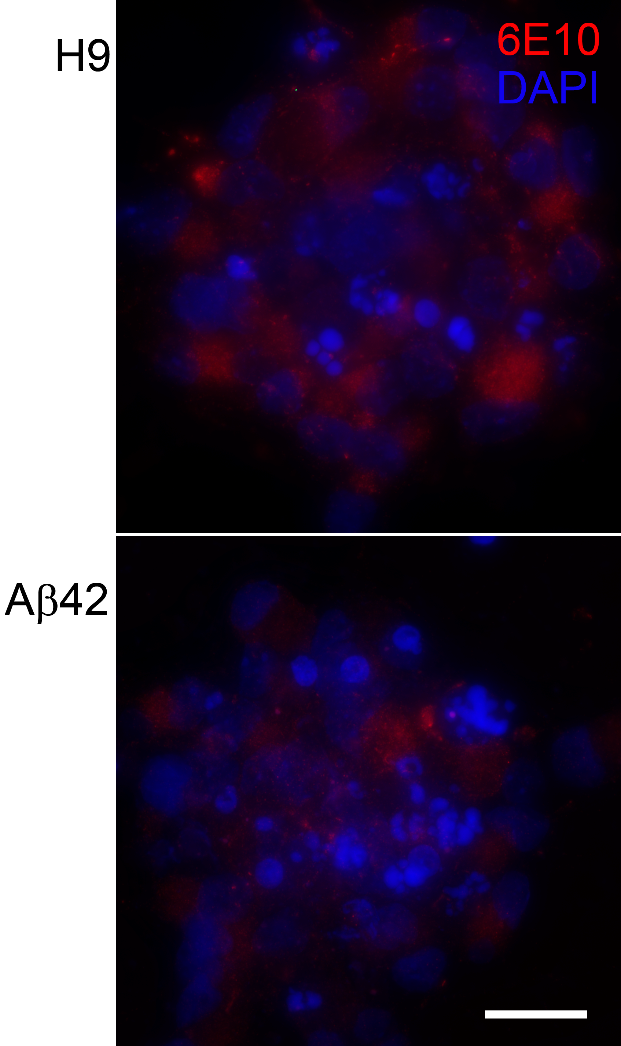


**Fig S5**. Immunocytochemical staining of Aβ in 32-day old cultures using antibody 6E10. There is apparently more Aβ accumulation in the cytoplasm in unedited H9 cells relative to Aβ 42edited cells at this stage. This suggests that this staining may be due to amyloidogenic processing of APP since the edited cells are heterozygous for APP. The brightness and contrast of the red channel has been adjusted the same for both images to show the comparative intensity of staining.


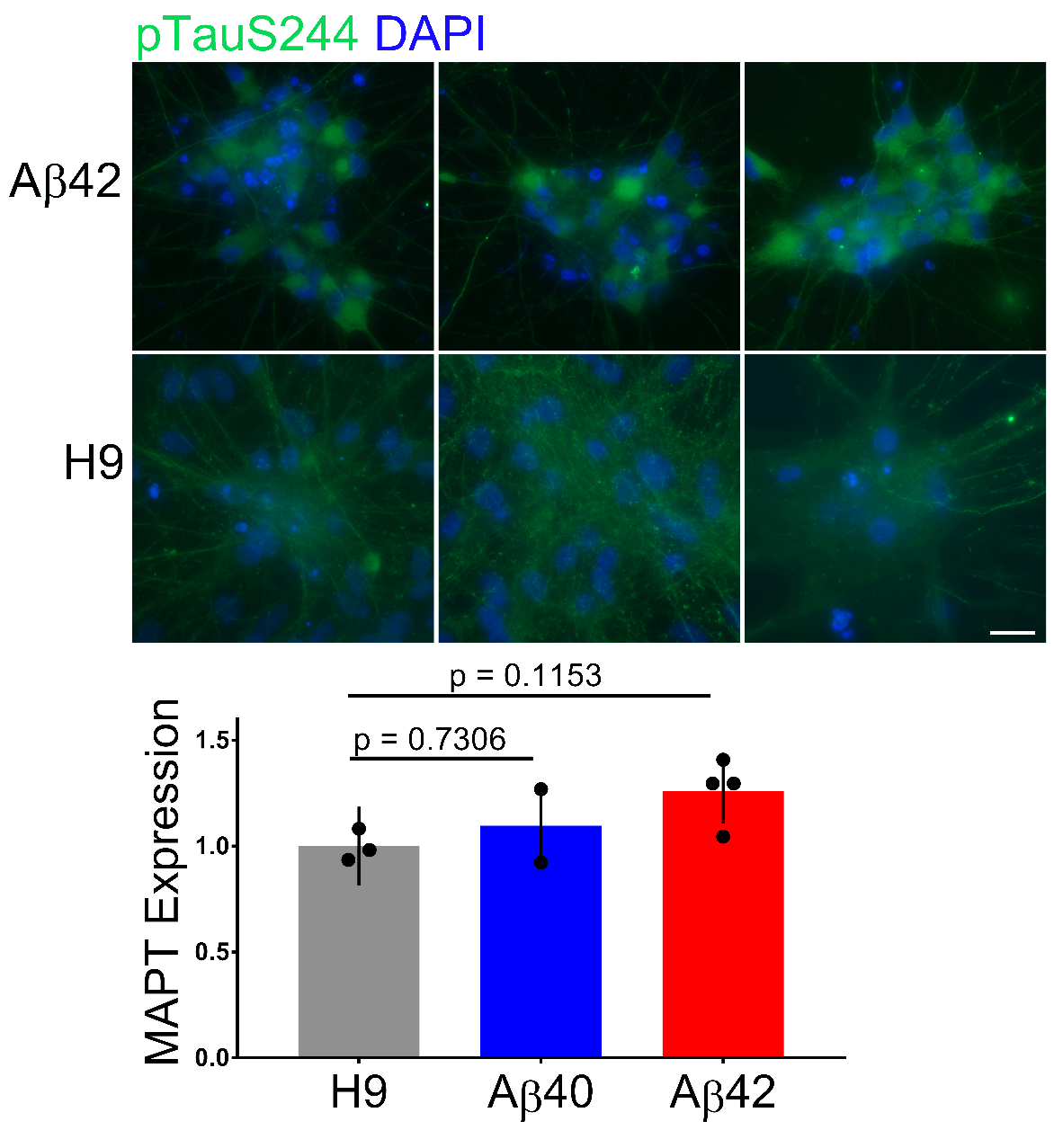


**Fig S6.** Older Aβ42 cultures show apparent distribution of phospho-tau in cell soma compared to H9 cultures where it is localized in neurites. (Top) Fluorescence images from 3 representative fields for each genotype taken from a 62-day old culture stained with anti-phospho-tau antibody (green) and DAPI (blue). This apparent difference is likely due to a significant decrease in neurites on dead or dying cells present in Aβ42 cultures rather than a redistribution of signal. The area of phospho-tau staining (normalized to DAPI) was not significantly different between H9 and Aβ42 samples (p=0.9078, N>15, t test). Scale bar = 20 μm. (Bottom), RNA-Seq analysis indicates no significant difference in relative MAPT expression (coding for tau) among the genotypes (ANOVA, Dunnett corrected). Data points are from independent RNA-Seq samples (±SEM).


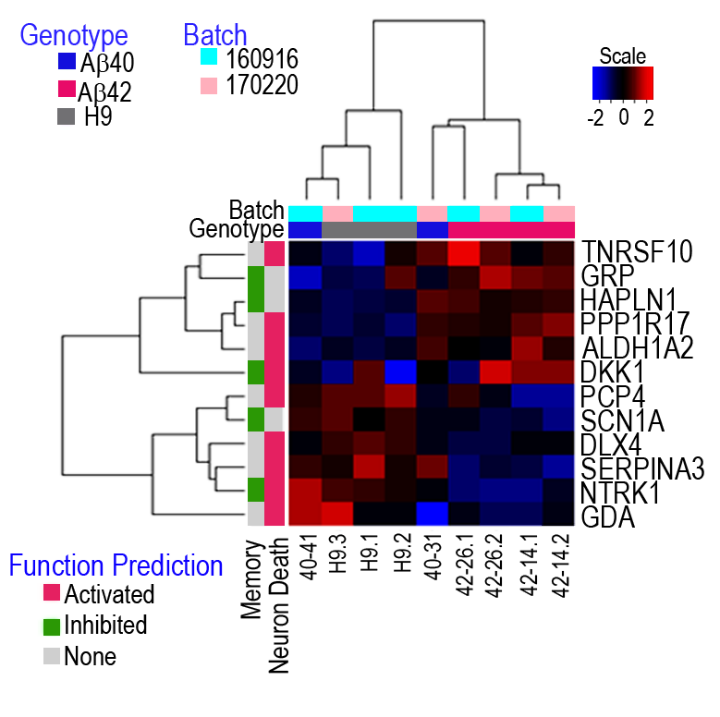


**Fig S7.** Clustered heat map showing relative sample level expression for IPA (Ingenuity Pathway Analysis) identified “Decreased Memory” and “Increased Neuronal Death” related genes.


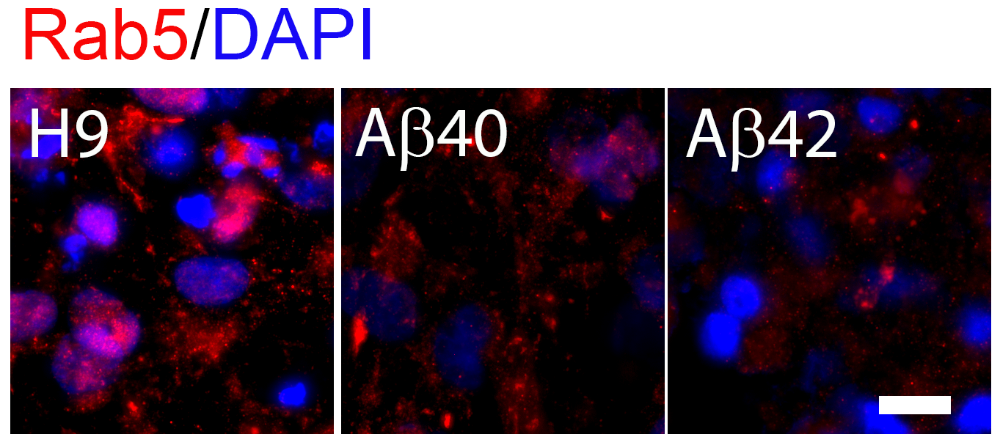


**Fig. S8.** Reduced endolysosomal vesicles in 63-day old Aβ40 and Aβ42 samples. The relative number of Rab5 puncta (normalized to DAPI) is reduced in later aged Aβ40 and Aβ42 cultures. Data is from individual NCs from 2 independent differentiations for 63-day cultures. Scale 10μm.

# References

1. Kim, D., et al., TopHat2: accurate alignment of transcriptomes in the presence of insertions, deletions and gene fusions. Genome Biology, 2013. **14**(4): p. 1-13.

2. Anders, S., P.T. Pyl, and W. Huber, HTSeq—a Python framework to work with high-throughput sequencing data. Bioinformatics, 2015. **31**(2): p. 166-169.

3. Hsu, F., et al., The UCSC Known Genes. Bioinformatics, 2006. **22**(9): p. 1036-1046.

4. Mortazavi, A., et al., Mapping and quantifying mammalian transcriptomes by RNA-Seq. Nat Meth, 2008. **5**(7): p. 621-628.

5. Lawrence, M., et al., Software for Computing and Annotating Genomic Ranges. PLOS Computational Biology, 2013. **9**(8): p. e1003118.

6. Robinson, M.D., D.J. McCarthy, and G.K. Smyth, edgeR: a Bioconductor package for differential expression analysis of digital gene expression data. Bioinformatics, 2010. **26**(1): p. 139-140.

7. Benjamini, Y. and Y. Hochberg, Controlling the false discovery rate: a practical and powerful approach to multiple testing. J R Stat Soc Series B Stat Methodol, 1995. **57**: p. 289-300.

8. Warden, C., Y. Yuan, and X. Wu, Optimal calculation of RNA-seq fold-change values. Int. J. Comput. Bioinformatics In Silico Model., 2013. **2**: p. 285–292

9. Ashburner, M., et al., Gene Ontology: tool for the unification of biology. Nat Genet, 2000. **25**(1): p. 25-29.

10. Young, M.D., et al., Gene ontology analysis for RNA-seq: accounting for selection bias. Genome Biology, 2010. **11**(2): p. 1-12.

11. Chang, J.T. and J.R. Nevins, GATHER: a systems approach to interpreting genomic signatures. Bioinformatics, 2006. **22**(23): p. 2926-2933.
